# Testing oral nicotine pouches versus nicotine replacement therapy for cigarette harm reduction in Appalachia: The ARISE study protocol

**DOI:** 10.1371/journal.pone.0338503

**Published:** 2025-12-23

**Authors:** Amy Wermert, Theodore M. Brasky, Alison M. Newton, Alice Hinton, Hayley Curran, Amy K. Ferketich, Matthew J. Carpenter, Peter G. Shields, Patrick Tomko, Theodore L. Wagener, Brittney Keller-Hamilton

**Affiliations:** 1 Center for Tobacco Research, The Ohio State University Comprehensive Cancer Center, The Ohio State University, Columbus, Ohio, United States of America; 2 Division of Medical Oncology, Department of Internal Medicine, College of Medicine, The Ohio State University Wexner Medical Center, Columbus, Ohio, United States of America; 3 Division of Epidemiology, College of Public Health, The Ohio State University, Columbus, Ohio, United States of America; 4 Department of Psychiatry and Behavioral Sciences, Medical University of South Carolina, Charleston, South Carolina, United States of America; PLOS: Public Library of Science, UNITED KINGDOM OF GREAT BRITAIN AND NORTHERN IRELAND

## Abstract

**Background:**

With the highest cancer incidence and mortality rates in the country, rural Appalachia has experienced a decades-long health decline, due in part to high smoking rates. Cigarette smoking prevalence exceeds 30% in much of the region. Oral nicotine pouches (ONPs), which contain nicotine but no tobacco, present an unexplored opportunity to reduce cigarette smoking and cancer incidence.

**Objectives:**

We outline the protocol for the Appalachian Research to Impact Smoking’s Effects (ARISE) study, a randomized controlled trial to determine whether ONPs affect cigarette smoking patterns short- and long-term, and to evaluate their abuse liability versus nicotine replacement therapy (NRT) in a large sample of Appalachian smokers (clinicaltrials.gov: NCT06763536).

**Methods:**

Between 2025 and 2029, we will recruit 1,000 adult smokers living in rural Appalachian counties across 11 states. Participants will be identified via media outreach, mobile cancer screening, community events, and respondent-driven sampling, then randomized to ONP or NRT and complete four study phases: Baseline, Sampling, Switch, and Observation. In the Sampling phase, participants will receive varied flavors and nicotine strengths of their assigned product and select preferred options for use. During the Switch Phase, they will attempt to quit smoking and switch completely to their assigned product. The Observation phase will monitor tobacco use after discontinuation of study products. Study procedures will be conducted online and by mail, including surveys, expired carbon monoxide verification, and product delivery. The primary outcome is 7-day biochemically verified cigarette abstinence at the end of the Switch Phase. Secondary outcomes include switching rates, product appeal, craving, withdrawal, dependence, and purchases during the Observation phase. An intention-to-treat log-binomial regression model will estimate the effect of intervention assignment on cigarette abstinence.

**Conclusions:**

Results will inform whether and how ONPs should be regulated, approached clinically, and used in public health interventions to reduce the burdens of cigarette smoking in Appalachia.

## Introduction

Over 26 million people live in the Appalachian region of the United States (US), which comprises 423 counties spanning from New York to Mississippi [1]. The region’s rural counties (approximately 25%) have been experiencing a decades-long decline in health, due in large part to persistently high smoking rates [2]. In the mid-20th century, rural Appalachia had the lowest cancer mortality rates in the US; now it has the highest cancer incidence and mortality rates [3,4]. This reversal is due to relatively indolent declines in cancer incidence and mortality in rural Appalachia relative to faster declines elsewhere in the US [3,5]. While most Appalachian cancer disparities have narrowed over time, disparities in incidence rates of head, neck, and lung cancers have widened [5]. These cancers share a common risk factor in tobacco use [6]. Whereas the prevalence of cigarette smoking is 11.5% nationally [7], the prevalence of cigarette smoking exceeds 30% in many rural Appalachian counties [2]. Accordingly, up to 40% of cancer deaths in Appalachian areas (vs. 29% in the US overall) can be attributed to cigarette smoking [8,9].

Cultural factors, like considering tobacco use a rite of passage, economic reliance on tobacco farming, and entrenched poverty and despair promote tobacco use in rural Appalachia [1,10–12]. Deficiencies in the rural Appalachian healthcare system, like lacking healthcare providers and health insurance, lead to reduced access to cessation services [13]. The tobacco industry’s actions also increased tobacco use in rural Appalachia. The industry has spent decades developing and manipulating tobacco products to appeal to new users, cultivate and maintain nicotine dependence, and increase the difficulty of cessation [14,15]. The industry additionally targeted Appalachia specifically with advertisements reinforcing Appalachian values of individuality and ruggedness [10,16].

Oral nicotine pouches (ONPs; e.g., ZYN, Velo, and On!), which entered the US consumer market in 2016 [17–19], present an as-of-yet unexplored opportunity to reduce cigarette smoking and cancer incidence. ONPs, which contain nicotine but no tobacco, have a toxicant profile similar to nicotine replacement therapy (NRT), with most carcinogens and toxicants below the limit of detection [20,21]. However, they are available in a wider range of nicotine concentrations and flavors than NRT, are marketed to smokers as spit-free and smoke-free alternatives to other tobacco products, and are often sold alongside cigarettes [22–24]. Crucially, ONPs are typically half as expensive as NRT lozenges [17–19,23]. We have previously shown that Appalachian smokers find ONPs to be more socially acceptable than cigarettes, more palatable than NRT, and that they reduce withdrawal symptoms similar to cigarettes [25,26]. It therefore stands to reason that ONPs might be a viable harm reduction option for Appalachian cigarette smokers.

We outline herein a randomized controlled trial to determine whether ONPs affect cigarette smoking patterns in the short- and long-term, and to evaluate their abuse liability relative to NRT in a large sample of Appalachian smokers. Excluding our preliminary studies [25–28], tobacco industry-sponsored studies examining ONPs’ appeal [29–32], and short-term pilot switching studies conducted in small samples [33,34], we are aware of no large, long-term, *independent* research comparing ONPs’ acute (e.g., withdrawal relief) or longer-term (e.g., cigarette abstinence) use effects among smokers vs. NRT. To our knowledge, this study will be the first independent investigation of how ONPs compare to combination NRT for cigarette smoking reduction and abstinence in rural Appalachia.

## Materials and methods

### Design overview

**Fig 1:**
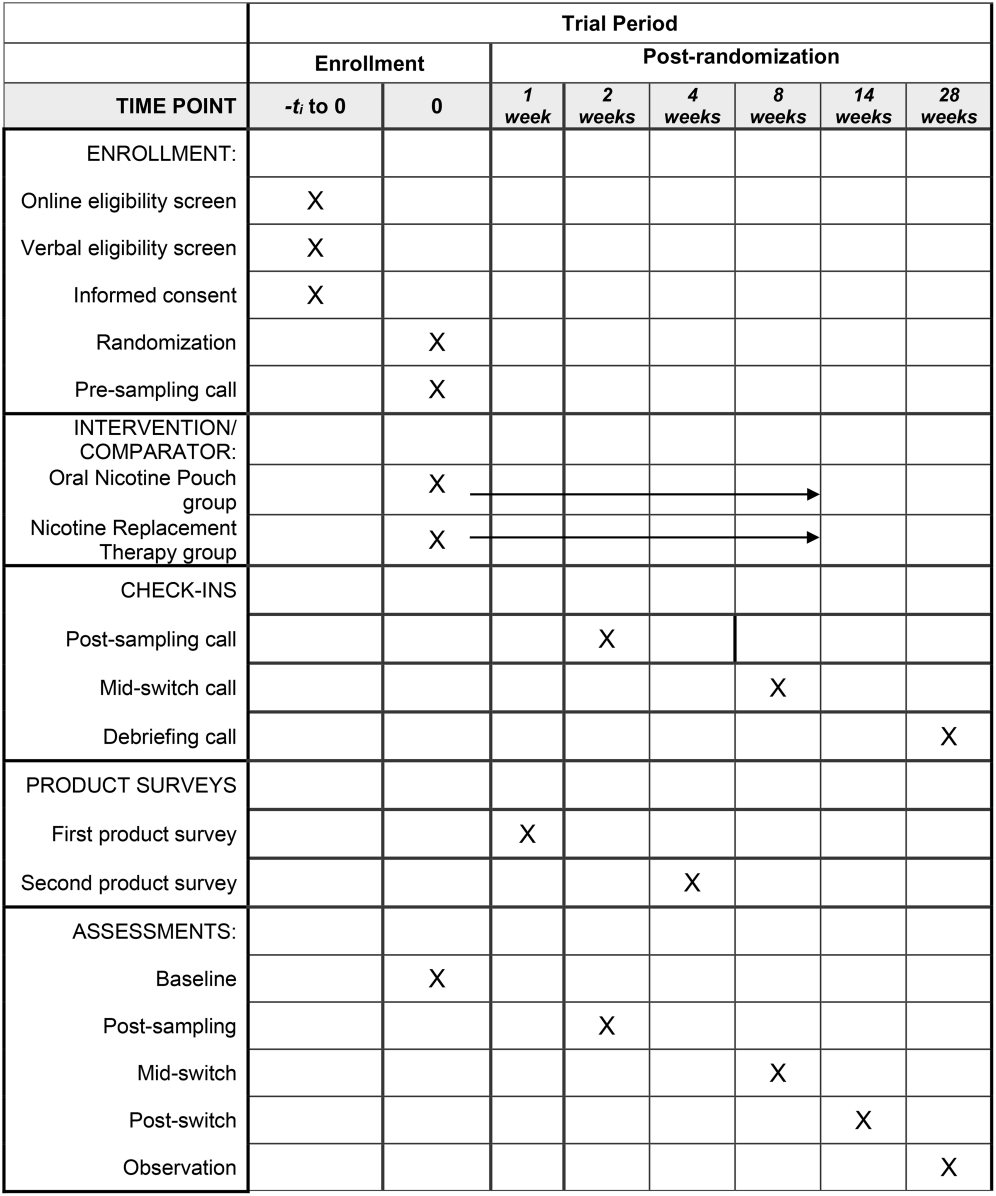
Standard Protocol Items: Recommendations for Interventional Trials (SPIRIT) participant timeline, schedule of enrollment, interventions, and assessments.

**Fig 2:**
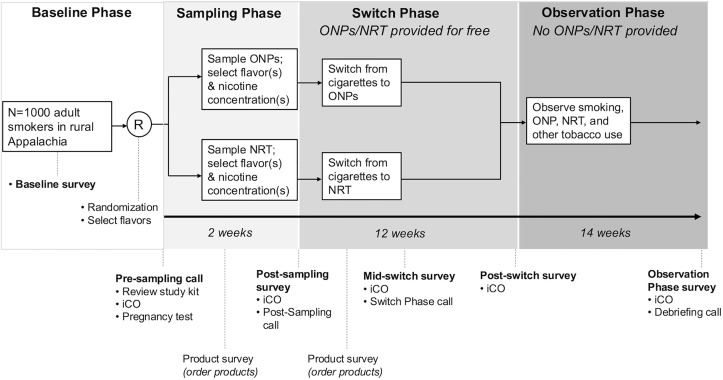
The ARISE trial of 1,000 adult rural Appalachian smokers, featuring the study’s four main phases: Baseline, Sampling, Switch, and Observation. During the Sampling and Switch phases, study products will be provided to participants at no cost. During the Observation phase, no products will be provided.

The Appalachian Research to Impact Smoking’s Effects (ARISE) study will be a 6-month randomized, parallel-arm, controlled switching trial of adult smokers living in rural Appalachia. ARISE encompasses 4 distinct phases: Baseline (pre-randomization), Sampling, Switch, and Observation (Figs 1–2). The study will be administered at The Ohio State University Comprehensive Cancer Center’s (OSU-CCC), Center for Tobacco Research in Columbus, Ohio. However, the trial will be conducted remotely online and by mail, without any in-person visits.

After randomization to the ONP or combination NRT (patch and lozenge) arm, during a 2-week Sampling Phase, participants will be sent ONPs or NRT in varied flavors and nicotine concentrations. Participants will familiarize themselves with their study product and will self-select nicotine concentrations and flavors to use for the Switch Phase. During the 12-week Switch Phase, participants will attempt to stop smoking cigarettes and switch entirely to their assigned study product. During the subsequent 14-week Observation Phase, participants will stop receiving study products, and their long-term switching behaviors will be monitored. Throughout the trial, self-reported changes in cigarette smoking via online surveys will be confirmed using remote expired carbon monoxide (CO) assessments.

### Objectives and hypotheses

The ARISE trial will address 3 primary objectives between participants assigned to ONP or NRT: 1) To evaluate short-term changes in cigarette smoking patterns, including switching, abstinence from cigarettes, and frequency of smoking; 2) To compare product appeal, craving, withdrawal relief, and perceived cigarette dependence; and, 3) To examine sustained changes in tobacco use behaviors, including abstinence from cigarettes, purchase of ONPs/NRT, and sustained use of ONPs/NRT.

Relative to the NRT group, we hypothesize that participants randomized to ONPs will: 1) have higher rates of complete switching (i.e., cigarette abstinence + study product use), greater abstinence from cigarettes, and smoke the fewest cigarettes per day during the 12-week Switch Phase; 2) report greater product appeal and greater reduction in craving and withdrawal symptoms; and, 3) have higher rates of abstinence from cigarettes, use their study product for a greater number of days, and be more likely to purchase their study product during the 14-week Observation Phase.

### Planned recruitment

Between June 13, 2025, and August 30, 2028, we plan to enroll 1,000 adult cigarette smokers, residing in 108 rural counties located across 11 Appalachian states: New York, Pennsylvania, Ohio, West Virginia, Virginia, Kentucky, North Carolina, Tennessee, Georgia, Alabama, and Mississippi. Following the Appalachian Regional Commission’s definition [1], we will use Urban Influence Codes (UIC), as defined by the US Department of Agriculture and Office of Management and Budget, to identify rural counties. Counties with a UIC > 6 (n = 108) will be prioritized for inclusion. If necessary, we will consider recruitment from counties with a UIC = 6 from these 11 states, as well as from Maryland (n = 69).

Potentially eligible individuals will be identified using a combination of community- and web-based approaches, including social media advertising, OSU-CCC mobile lung cancer screening, outreach to community organizations, contacting participants from existing studies, newspaper advertisements, and respondent-driven sampling [35,36].

Interested adults will initially be screened for eligibility using an online questionnaire, and then again as part of a video call (phone call will be offered to participants with limited technology expertise or weak wireless internet), during which additional study details will be provided. Those meeting the following eligibility criteria will be asked to participate: 1) adults ≥21 years of age; 2) have smoked ≥5 cigarettes per day for the past year; 3) willing to use oral nicotine pouches or nicotine replacement therapy; 4) live in a rural Appalachian County; 5) are not breastfeeding, pregnant, or planning to become pregnant within 6 months; 6) have a smartphone and/or be willing to use a smartphone; 7) willing to use a handheld study device for expired CO testing; and 8) read and speak English. Exclusion criteria include: 1) report currently using smoking cessation medications, NRT, or actively seeking treatment for smoking cessation; 2) use of ONPs or NRT in the past three months; 3) use of tobacco products other than cigarettes >10 days/month; 4) self-reported unstable or significant medical condition; 5) self-reported unstable or significant psychiatric conditions (past and stable conditions will be allowed); 6) self-reported history of cardiac event or distress within the past 3 months; and 7) live in same household as another study participant. Upon confirmation of eligibility, participants will provide informed consent via an electronic form.

### Procedures

#### Baseline Phase.

At the conclusion of the Orientation Call, participants will receive a link to the baseline survey. This survey will include questions on tobacco use history as well as the perceived risks and benefits of tobacco products, nicotine dependence and withdrawal scales, quit intention and motivation to quit, tobacco use in the household and among social circles, alcohol and cannabis consumption, general health and respiratory health status, and sociodemographics including participants’ dates of birth, sex, race/ethnicity, education, employment and household income, and marital status (Table 1 and S1 Table). Cigarette consumption over the past week will be assessed using a timeline follow-back method [37]. Participants will first review their daily activities and routines over the previous seven days, which will serve as memory anchors. Using these cues, they will then report their cigarette use each day, allowing for more accurate recall than generic daily estimates [37]. Participants will also be asked which NRT and ONP flavors and nicotine concentrations they would like to sample and will be permitted to select up to 6 varieties of ONPs or lozenges (details below). After completing the baseline survey, participants will be stratified by sex, ONP use history (ever/never), and self-reported cigarette dependence (Fagerström Test for Nicotine Dependence <5/ ≥ 5), and randomized to either the ONP or combination NRT arms for 14 weeks by the study’s biostatistician (Dr. Hinton), encompassing the Sampling (2 weeks) and Switch (12 weeks) phases of the trial. Randomization will be performed unblinded using SAS v.9.4 and implemented via the Research Electronic Data Capture (REDCap) platform. The randomization sequence will not be made available to any study investigators or staff.

**Table 1:** Selected ARISE study measures.

Measure	Baseline^1^	Sampling Phase		Switch Phase		Observation
		(2 weeks)		(12 weeks)		(14 Weeks)
		Pre-Sampling Call^2^	Post- Sampling Survey^3^	Mid- Switch Survey^4^	Post-Switch Survey^5^	Observation Survey^6^
Socio-demographics/environment	X					
Tobacco & NRT Use History	X					
Current Alcohol and Marijuana Consumption	X					
Smoking urges, nicotine withdrawal and Nicotine Dependence	X		X	X	X	X
Smoking cessation history, motivations and attitudes	X		X	X	X	X
Health questionnaire	X		X	X	X	X
Current Use and Experience with usual brand	X		X	X	X	X
Perceived Risk / Opinions – Cigarettes, ONPs, NRT	X		X	X	X	X
Current tobacco, ONP and NRT Use			X	X	X	X
Study product experience, perceptions, dependence			X	X	X	X
Study Product Adherence				X	X	X
Intentions to use study product after the study					X	X
Quitting Nicotine (interest in quitting)						X
Product Transitions (use of ONP and NRT not provided by the study)						X
Adverse Events			X	X	X	X
iCO measurement and CO questions		X	X	X	X	X

^**1**^ Day 1

^**2**^ After study kit arrives

^**3**^ Prior to post-sampling call

^**4**^ 6 weeks after switch date

^**5**^ 12 weeks after switch date

^**6**^ 14 weeks after switch date

**Fig 3:**
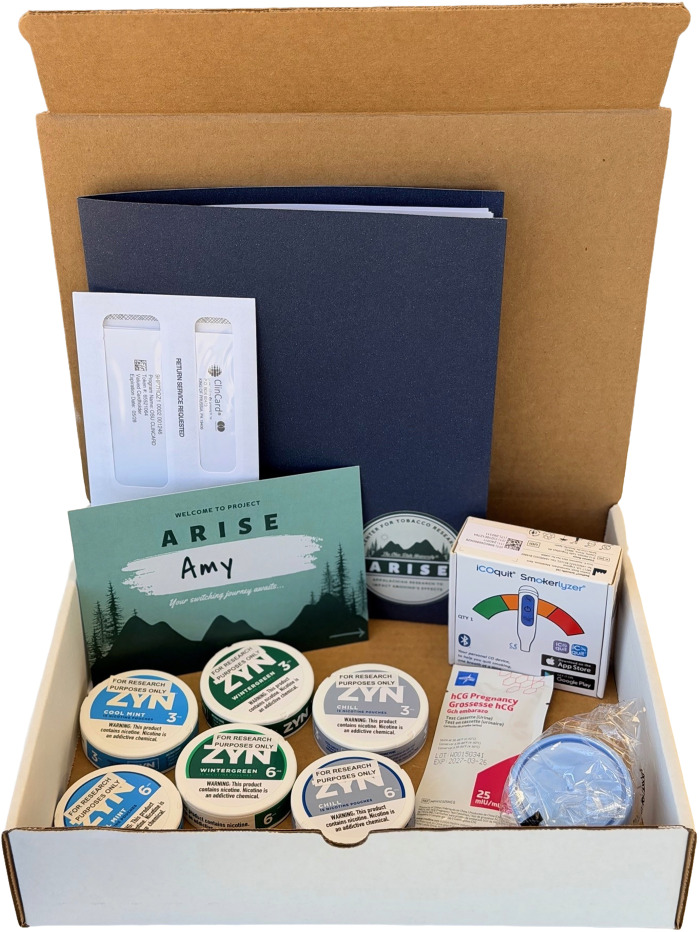
An example ARISE trial sampling kit for a participant randomized to the oral nicotine pouch intervention.

Following randomization, participants will be mailed their study kits, tailored to group assignment. All participants will receive study kits that will include a welcome card and letter of thanks, a copy of the consent form, a nicotine overdose fact sheet, participant roadmap outlining their involvement in the study, a Bedfont iCOquit Smokerlyzer device and instructions, a pregnancy test for those who can become pregnant (i.e., pre-menopausal female participants who have not undergone irreversible sterilization methods such as tubal ligation), and study products to sample (Fig 3). Participants will be asked to avoid smoking cannabis products in the 24 hours prior to CO measurements. Participants will also receive a ClinCard for participant incentives (see Participant Incentives, below) and instructions on how to use it, and information about the referral program for peer recruitment. Study product fact sheets, product guidelines, and product troubleshooting tips specific to study group will additionally be provided.

For the initial 2-week sampling phase, participants randomized to the ONP group will receive three cans (15 pouches) of ZYN oral nicotine pouches in up to three flavors at the 6 mg concentration and three cans in up to three flavors at the 3 mg concentration, based on the flavor preferences indicated in the baseline survey. Choices of flavors will include cool mint, citrus, peppermint, wintergreen, spearmint, and chill (unflavored) contingent upon their market availability at the start and duration of the trial. The choice of ONP brand and flavors was determined by market share. Participants randomized to the NRT group will receive one box each of 14 mg and 21 mg Rugby brand nicotine patches (14 to a box), as well as three tubes of GoodSense brand nicotine lozenges in up to three flavors at the 2 mg concentration and three tubes of 4 mg nicotine concentration in up to three flavors; number of lozenges per tube vary by flavor), based on their selections from the baseline survey. Lozenge flavor choices will include mint, ice mint, and cherry. All products will be given to participants in their original packaging and at no cost. For both ONPs and lozenges, new flavors may be offered to participants if existing flavors are discontinued or if new flavors become available. Product packaging will be clearly labelled “For Research Purposes Only,” and participants will be instructed to store the products away from children and pets.

#### Sampling Phase (2 weeks).

The goal of the Sampling Phase is for participants to become familiar with the study product, as product sampling is particularly important in supporting smoking cessation success among rural smokers [38,39]. A second goal of the Sampling Phase is for participants to choose the nicotine concentration(s) and flavor(s) they will attempt to switch to during the first half of the Switch Phase. Participants will be instructed not to begin using their products until after completing a pre-Sampling call with study staff, which will include a full review of the study kit including study product education, use instructions, and troubleshooting. During the call, participants will confirm a negative pregnancy test and complete their first CO measurement to confirm their smoking status. Participants with CO measurements <7ppm will be unable to participate further. Participants will set an agreed upon switch date (≥2 weeks later) and will be asked to immediately start using their sample products *ad libitum*.

One week into the Sampling Phase, participants will complete a brief survey to record their preferred product concentration(s) and flavor(s) for use in the upcoming Switch Phase. Ten days after beginning the Sampling Phase, participants will complete a post-Sampling survey that evaluates their confidence in switching to their study product and their experiences and sampling frequency during the Sampling Phase (Table 1). A timeline follow-back for past week use of cigarettes, ONP, and NRT will also be completed, and participants will again be asked to take a CO measurement. Participants will be sent their first kit of products for the Switch Phase, based upon the number of cigarettes smoked per day as reported at baseline (1.5 ONPs or NRT lozenges per cigarette smoked per day and a 6-week supply of NRT patches). Approximately 2 days prior to the participant’s switch date, there will be a post-sampling call with study staff to confirm receipt of additional products for the first six weeks of the Switch Phase, a reminder of participants’ chosen switch date, and that upon waking the morning of their switch date they should not smoke cigarettes. An email will be sent if a call cannot be made. They will be instructed to stop smoking and continue with their allocated study product (ONP or NRT) for 12 weeks.

#### Switch Phase (12 weeks).

The goal of the Switch Phase is to identify whether there are differences in cigarette smoking patterns (i.e., switching to the study product, abstinence from cigarettes, and frequency of smoking) between the assigned groups. We will also assess potential differences in subjective measures of product appeal, craving and withdrawal relief, and changes in perceived cigarette dependence between groups.

The Switch Phase will begin on participants’ chosen switch dates and last for 12 weeks. At week four of the Switch Phase, participants will complete a brief survey to report their preferences for nicotine concentration(s) and flavor(s) for the study products they wish to use for the final 6 weeks of the Switch Phase. The second kit of products will be determined based upon the number of cigarettes smoked per day, the number of study products used per day, and the approximate count of study products they have remaining in stock. Upon receipt of study products, study staff will conduct a check-in call with participants to answer any product- or study-related questions. If staff cannot connect, an email will be sent. There will be surveys at 6 (mid-switch) and 12 weeks (post-switch) after the switch date. The surveys will include timeline follow-backs for use of cigarettes, ONPs, and NRT, and the post-switch survey will assess product holdover. Expired CO measurements will also be taken and recorded. Participants will additionally be asked questions to assess product appeal, craving and withdrawal, and perceived nicotine dependence (**Table 1**).

#### Observation Phase (14 weeks).

The Observation Phase will begin immediately after the Switch Phase and will last for 14 weeks. The goal of the Observation Phase is to assess changes in smoking and sustained use of ONPs/NRT when they are no longer provided cost-free. Secondary goals will include assessing changes in nicotine concentrations, flavors, and brands used, as well as uptake of the other nicotine/tobacco products. At the end of the Observation Phase, participants will again complete a past week timeline follow-back assessment to measure use of cigarettes, ONPs, and NRT. They will additionally answer questions on perceived nicotine dependence and intent to use ONPs/NRT after the study, and a CO measurement will be taken. After the survey is submitted, participants will receive a debriefing call during which those who report continued cigarette smoking will be strongly advised to quit smoking and will be emailed cessation resources. Those who have completely switched to ONPs or NRT will be encouraged to continue cigarette abstinence and will be emailed cessation resources to assist with quitting all nicotine.

### Participant incentives

Participants will be provided with a ClinCard prepaid Mastercard for incentives. $25 will be loaded to the participant’s ClinCard for each survey/assessment completed and a $25 bonus will be added for completing all 5 assessments. Participants may also receive $10 for each cigarette smoker they refer (up to 5) who completes the online and verbal screenings. The maximum total incentive will be $200.

### Primary outcomes

Biochemically verified 7-day point prevalence of abstinence from cigarettes at the end of the 12-week Switch Phase by study arm is the primary outcome of the study and was chosen because it is a critical outcome to inform public health interventions and potential FDA regulations of ONPs. Data will be obtained from the post-switch self-reported timeline follow-back measure and its corresponding CO measurement (≤6 ppm) for verification.

### Secondary outcomes

The study has several secondary outcomes contrasting participants randomized to the ONP and NRT groups, including differences in: 1) product use; 2) dependence, withdrawal, and craving; and 3) product appeal. Product use: Using timeline follow-backs and biochemical verification, we will assess 7-day point prevalence of abstinence from cigarettes at the end of the Observation Phase; 7-day complete switching (cigarette abstinence + daily study product use over the past 7 days) at the end of the Switch and Observation Phases; past 7-day frequency of study product use at the end of the Switch and Observation Phases; changes in the frequency of cigarettes used per day from baseline to the end of the Switch and Observation Phases; and purchase of study products during the observation phase. Dependence, withdrawal, and craving: At the end of the Switch and Observation Phases, we will assess differences in cigarette dependence (e.g., cigarette dependence scale, FTND, and Penn State Cigarette Dependence Index). We will also examine differences in cigarette and nicotine dependence (e.g., Penn State Cigarette Dependence Index, PROMIS nicotine dependence scale), cigarette craving (e.g., Questionnaire on Smoking Urges-Brief, craving item-Minnesota Nicotine Withdrawal Scale), and nicotine withdrawal (e.g., Minnesota Nicotine Withdrawal Scale) at the end of both phases. Product appeal: Lastly, we will examine differences in ONP and NRT product appeal at the end of the Switch Phase (e.g., modified cigarette evaluation questionnaire, study product effects scale).

## Analysis

Data collection will be completed for all participants by April 30, 2029 with primary results anticipated by August 31, 2029. Only participants who complete the baseline assessment, biochemically verify themselves as a smoker at the pre-sampling call, and confirm they are not pregnant (if applicable), will be included as part of an intention-to-treat (ITT) analysis. If a participant drops out, we will censor data at the point of loss. Primary analyses will follow a conservative ITT approach where participants with missing data will be assumed to still be smoking. If necessary, either due to an unexpected amount of missing data or a missingness pattern imbalanced between the groups, the ITT approach will be compared to one in which inverse probably weighting with propensity scores is used. The probability of missingness will be modeled as a function of baseline covariates and previous outcomes; the inverse of the resulting predicted probabilities will then serve as weights in our proposed model of the response.

Statistical analyses will be performed using SAS 9.4. *P*-values<0.05 will be considered statistically significant. Baseline demographics and participant characteristics will be summarized by arm (ONP and NRT), as appropriate. Continuous variables will be presented as mean ± standard deviation and compared between the arms with t-tests. Categorical variables will be presented as frequencies and proportions and compared with chi-squared tests. The balance of all covariates according to randomization will be confirmed prior to conducting the below analyses. Any imbalanced covariates will be controlled for in regression models.

Log-binomial regression models, adjusting for baseline variables as needed, will be used to examine differential probabilities of abstinence from cigarettes at the end of the Switch Phase. Examination of secondary product use, product appeal, and dependence, withdrawal, and craving outcomes will be performed using a set of generalized linear models with appropriate link functions (e.g., identity for continuous outcomes, log link for binary outcomes). Normalizing transformations of continuous outcomes will be employed as appropriate for violations of residual normality or homoscedasticity. For examination of cigarettes used per day, a Poisson or negative binomial regression model will be fit, adjusting for baseline cigarette use frequency if found to be imbalanced by randomization. We will additionally explore whether participants‘ sex modifies the associations between study arm and each outcome using interaction terms in regression models. Adverse events (AE) and serious adverse events (SAE), outlined below, will be summarized and differences between the arms tested using a chi-squared test.

### Sample size and power

Based upon our prior trial that investigated the effects of NRT sampling on cessation outcomes [39], we observed a cigarette abstinence rate of 13% among rural participants in the NRT arm. In a separate pilot crossover trial of two doses of ONP [25], 23% of participants indicated in a 6-month post-trial follow-up survey that they abstained from cigarettes (unpublished results). Using these estimated abstinence rates of 13% and 23% for NRT and ONP, respectively, and assuming 80% retention of the recruited sample (n = 1,000) at the end of the 12-week Switch Phase, a total of 800 participants, will provide >95% power to detect a difference in smoking abstinence between the trial arms for a two-sided, α = 0.05 level chi-squared test. Assuming 70% retention at the end of the 14-week Observation Phase, a sample of 700 will provide >90% power to detect a significant difference in abstinence between the ONP and NRT arms.

### Data management

All study materials will be kept on the OSU-CCC’s secure computer network behind a firewall and password protected. Study data will be collected, managed, and secured using the REDCap platform, which will be accessible to authorized study personnel. All computer systems will be password-protected to prevent unauthorized access, and all network-based inter-site communications of confidential data will be encrypted. Participants will be assigned a unique identifier which will be used to identify them in all study data. Participant information will be accessible only to authorized research staff who are pledged to confidentiality and have received training in the ethical conduct of research.

## Safety considerations

### Potential risks

This trial will be conducted in a sample of adults who smoke combustible cigarettes. The study products, including the ONP intervention and the combination NRT control, are available over the counter and are anticipated to represent low risk to participants. ONPs are not combusted and do not contain tobacco leaf, and as a result, they carry substantially lower levels of toxicants than cigarettes [21]. A recent study reported adverse events following administration of ONP, NRT lozenge, and NRT gum (4 mg of nicotine each) [32]. The incidence of product-related adverse events was lowest in the ONP group (12.1%) followed by NRT gum (12.5%) and NRT lozenge (30.3%). Overall, 97% of AEs were mild, with 6% of participants reporting dizziness after ONP use (the most common AE) and no reports of nausea or throat irritation following ONP use. No serious AEs were reported in the ONP group. AEs from combination NRT have been well-characterized over years of study [40–46]. The most common anticipated AEs from lozenges include nausea, mouth and throat irritation, and hiccups [42]. The most common AEs from patches include skin irritation, insomnia, and headache or nausea [42,44,45].

### Protections against risks

At baseline, potentially eligible participants will be screened for pregnancy (or intention to become pregnant) and other medical contraindications for participation and removed from eligibility. Participants will complete AE questionnaires at each survey, including at baseline to establish background information to evaluate later-reported AE symptoms. Study staff will also complete an AE questionnaire with participants if they contact staff to report new symptoms. The AE questionnaire will assess common nicotine-related symptoms (e.g., headache, nausea, sleep disturbances, throat/mouth irritation) as well as more serious events (e.g., abnormal heartbeat, seizures, difficulty breathing), with participants rating whether symptoms were absent, present but not requiring medical attention, or present requiring medical attention. Study staff will review AE surveys and reach out to participants if more information is needed. AEs rated as at least “moderate” and possibly related to the study product will be routed to the medical monitor (Dr. Shields) and MPIs (Drs. Keller-Hamilton and Wagener) for review. The medical monitor will provide final ratings of severity and seriousness, as well as attribution to the study product. Any SAE will be promptly reported to The Ohio State University’s Institutional Review Board (IRB), and the National Cancer Institute. Participants who experience an AE/SAE or a change in health status related or unrelated to study product use may be withdrawn from the study per advice of the medical monitor. Upon study completion, participants will be encouraged to cease smoking and/or quit nicotine.

## Data sharing and dissemination

We will share de-identified individual-participant level (IPD) data. Appropriate measures such as removing all personally-identifiable information will be used for data de-identification and sharing, and informed consent forms will reflect those plans. To facilitate interpretation of the data, data dictionaries and codebooks will be created, shared, and associated with the relevant datasets. Documentation and support materials will be compatible with the clinicaltrials.gov Protocol Registration Data Elements, where applicable. All dataset(s) that can be shared will be deposited in the Inter-university Consortium for Political and Social Research (ICPSR). ICPSR will make the research data from this project available to the broader social science research community as public-use data files. These files, in which direct and indirect identifiers have been removed to minimize disclosure risk, may be accessed directly through the ICPSR website. The research data from this project will be supplied to ICPSR at the time of publication of the relevant results or at the end of the project period, whichever is sooner, so that any issues surrounding the usability of the data can be resolved.

The study team’s plan for dissemination targets researchers, clinicians, participants, and the public. The results of this trial will be presented at national or international scientific conferences and submitted for publication in refereed journals. Results will be promptly reported to clinicaltrials.gov. The study team will work with the OSU-CCC’s public relations department to develop and send out press releases describing results that may be of interest to the public. We will additionally disseminate findings on professional listservs read by researchers and clinicians with an interest in ONPs’ effects. Lastly, participants will be asked on the final survey whether they would like to receive a brief summary of the study’s results in lay language after the trial’s conclusion.

## Ethical declarations

This study is registered with clinicaltrials.gov under the identifier NCT06763536, submitted 1/2/2025. The study’s protocol has been reviewed and approved by OSU’s Cancer IRB under Study Number 2024C0064 on 6/3/2024 (modification version 2 approved 9/15/2025). Informed consent, including a legally valid signature, will be obtained using both verbal and written descriptions of the study and REDCap system. Participants will be clearly informed that their participation is voluntary and that they may withdraw at any time. An electronic signed copy of the consent form will be provided to participants at the time of consent for their records. Only IRB-approved study personnel will be responsible for obtaining consent. Any modifications to the study protocol will be submitted to the OSU Cancer IRB and ClinicalTrials.gov, and such changes will be documented in future publications as necessary.

## Discussion

Rural Appalachia is home to a staggeringly high prevalence of cigarette smoking compared to the rest of the US [2]. Unsurprisingly, this increased smoking burden translates to elevated incidence of lung and oral cancers [5]. With cultural-, healthcare system-, and tobacco industry-related factors bolstering smoking in rural Appalachia and limited access to cessation treatments, we propose to investigate whether new, lower-risk ONPs can be used to reduce the health burdens of cigarette smoking in this region. Ultimately, the results of this study will inform how ONPs should be regulated, approached clinically, and used in public health interventions to reduce the burdens of cigarette smoking in rural Appalachia.

We anticipate the following limitations of the ARISE study. First, because this study will be conducted among adults who live in rural Appalachian counties of the US, we cannot expect that results will generalize to other populations inside or outside of the US. Second, due to storage space and shelf-life limitations, we cannot purchase all study products at once. This means that study products in both arms might change in minor ways over time due to batch effects. Perhaps more concerningly, it is also possible that study products in both arms will change over time due to products being discontinued. For example, specific flavors or nicotine concentrations of ONPs, nicotine lozenges, or nicotine patches might be removed from the market over the course of the study. If this should happen, we plan to replace the discontinued study product variety with a new one, assuming a replacement product is available. A third and related limitation is that fewer flavors are available for nicotine lozenges than for ONPs. Although we attempted to match the number of flavors, we were unable to find a nicotine lozenge vendor that could fulfill our large order sizes in more than three flavors. Finally, our biochemical confirmation of changes in smoking status, expired CO, would not detect instances of participants switching to a different non-combustible nicotine product, like an electronic cigarette or smokeless tobacco.

The ARISE study also has several strengths. First, investigating the utility of ONPs for smoking reduction in rural Appalachia will have a large impact on public health because the prevalence of smoking in this region remains disproportionately high. A second way that our study could have a large impact on public health is related to our intervention product. ONPs are readily available at gas stations, convenience stores, grocery stores, and online [47,48]. ONPs are also a relatively inexpensive product in the larger nicotine and tobacco market. Both the availability and affordability of ONPs position them well for a scalable intervention to reduce cigarette smoking if we find that ONPs improve smoking reduction outcomes. Finally, our large sample size (n = 1,000) is well-powered to detect statistically significant effects of the intervention on our primary and secondary outcomes.

In conclusion, the ARISE study will compare ONPs vs. combination NRT for short- and long-term outcomes of smoking reduction, cigarette abstinence, complete switching, and subjective effects in a large sample of rural Appalachian cigarette smokers. This study will provide results that can be used to improve public health regardless of whether our hypotheses are supported. If, as expected, we find that using ONPs increases the probability of smoking abstinence (vs. NRT), results will be useful to public health practitioners, regulators, and healthcare providers who are working to reduce smoking in rural Appalachia. If we find that ONPs perform similarly (or worse) than NRT on smoking outcomes, results will provide evidence that ONPs provide little public health benefit, which will be useful to regulators considering whether it is appropriate that they remain on the market as tobacco products. Thus, the ARISE study is poised to have a significant effect on public health.

## Supporting information

S1 Protocol ARISE Protocol v2 no pictures. (DOCX)

S1 Checklist SPIRIT 2025 Editable Checklist. SPIRIT 2025 checklist of items to address in a randomized trial protocol.* *We strongly recommend reading this checklist in conjunction with the SPIRIT 2025 Explanation and Elaboration and the SPIRIT 2025 Expanded Checklist for important clarifications on all the items. We also recommend reading relevant SPIRIT extensions. See www.consort-spirit.org. Citation: Chan A-W, Boutron I, Hopewell S, Moher D, Schulz KF, et al. SPIRIT 2025 statement: updated guideline for protocols of randomised trials. BMJ 2025;389:e081477. https://dx.doi.org/10.1136/bmj-2024-081477. © 2025 Chan A-W et al. This is an Open Access article distributed under the terms of the Creative Commons Attribution License (https://creativecommons.org/licenses/by/4.0/), which permits unrestricted use, distribution, and reproduction in any medium, provided the original work is properly cited.
(DOCX)

S1 Table ARISE study measures. (DOCX)

## Abbreviations

AE: Adverse event
ARISE: Appalachian Research to Impact Smoking’s Effects
CO: Carbon monoxide
NRT: Nicotine replacement therapy
ONP: Oral nicotine pouch
OSU-CCC: The Ohio State University Comprehensive Cancer Center
REDCap: Research Electronic Data Capture
SAE: Severe adverse event
SPIRIT: Standard Protocol Items: Recommendations for Interventional Trials
UIC: Urban influence code
US: United States
