# Supplementary material for: Testing oral nicotine pouches versus nicotine replacement therapy for cigarette harm reduction in Appalachia: The ARISE study protocol
Source: PLoS One. 2025 Dec 23;20(12):e0338503. doi: 10.1371/journal.pone.0338503 (PMC12725661; doi:10.1371/journal.pone.0338503)
Supplement: S1 Protocol — (DOCX) [file pone.0338503.s001.docx]

**Testing Oral Nicotine Pouches to Reduce Smoking-Related Cancer Disparities in Rural Appalachia**

**Principal Investigator:** Brittney Keller-Hamilton, PhD, MPH

Medicine/IM Medical Oncology | Center for Tobacco Research

3650 Olentangy River Rd, Suite 420 Columbus, OH 43214

614-366-9652

Brittney.Keller-Hamilton@osumc.edu

**Sponsor**: National Cancer Institute (NCI)

**Funder**: National Cancer Institute (NCI)

**Drug/Device**

**Manufacturer**: N/A

**IND Number**: N/A

**IDE Number**: N/A

**Initial version:** V 1.0 [6/14/2024]

**Amended:** V 2.0 [date]

# **Table of Contents**

1. **Background and Rationale**
2. **Objectives**
3. **Study Design & Procedures**
4. **Institutional Approvals**
5. **Participant Population**
6. **Cost to Participants and Incentives to Participate**
7. **Compensation for Research-Related Injury**
8. **Informed Consent Process**
9. **Privacy of Participants**
10. **Confidentiality and Management of Study Materials**
11. **HIPAA Research Authorization**
12. **Reasonably Anticipated Benefits**
13. **Risks, Harms, & Discomforts**
14. **Monitoring**
15. **Data Analysis**
16. **Bibliography**
17. **Appendix**

# **1. Background and Rationale**

**Introduction**

The lung and oral cancer disparities that plague Appalachia are fueled by cigarette smoking. Over 26 million people live in the Appalachian region of the United States (US), which comprises 423 counties spanning from New York to Mississippi. ^8^ Rural counties in the Appalachian region (42% of counties) have been experiencing a decades-long, relentless decline in health, due in large part to persistently high smoking rates. ^4^ Following World War II, rural Appalachia had the lowest cancer mortality rates in the US; now, rural Appalachia has the highest cancer incidence and mortality rates.^2,3^ This reversal is due to relatively indolent declines in cancer incidence and mortality in rural Appalachia in comparison to faster declines elsewhere in the US.^1,2^ While most rural Appalachian cancer disparities have narrowed over time, disparities in incidence rates of larynx, oral cavity and pharynx, and lung and bronchus cancers have been growing. ^1^ These cancers share a common risk factor, tobacco use,^19^ which is a more prevalent behavior in rural Appalachia than elsewhere in the US.^20,21^ Nationally, the prevalence of cigarette smoking is 11.5%.^5^ In many rural Appalachian counties, the prevalence of cigarette smoking exceeds 30%. ^4^ Accordingly, up to 40% of cancer deaths in Appalachian areas (vs. 29% in the US overall) can be attributed to cigarette smoking.^22,23^

Cultural- and healthcare system-level factors bolster cigarette smoking in rural Appalachia. Cultural factors that support tobacco use in rural Appalachia include social norms permissive of adolescent tobacco use in the home;^7^ considering tobacco use a rite of passage and a way to emulate older family members;^6^ and dual use of oral tobacco while coal mining due to cigarette smoking being unsafe.^7^ Opposition to tobacco control policies in rural Appalachia, driven by economic reliance on tobacco farming, libertarian values, and distrust of “outsiders” forcing their policy agendas on local communities, also drive tobacco use.^7,24,25^ Consequently, people living in rural Appalachian communities are less likely to have smoke-free policies at worksites, restaurants and bars, and public parks than those living in urban areas.^26,27^ States with large rural and/or rural Appalachian populations also have lower cigarette excise taxes in comparison to the state average of $1.91/pack^28,29^  and are less likely to have tobacco retailer licensing laws,^30^ inhibiting a state’s ability to enforce retailer compliance with tobacco control policies. Deficiencies in the rural Appalachian healthcare system also contribute to an increased prevalence of cigarette smoking.^10,31,32^ Although rates of health insurance coverage in Appalachia generally align with the US national average^8^ and participation in Appalachian smoking cessation programs increase the probability of successful cessation,^33^ health care providers are lacking. Overall, the Appalachian region has 12% fewer primary care physicians per 100,000 population than the rest of the US.^31^ The supply of primary care physicians is even lower in rural Appalachian counties, which have 20% fewer primary care physicians per 100,000 population than urban Appalachian counties.^31^ With reduced primary care utilization, rural Appalachian smokers have fewer opportunities to benefit from brief cessation counseling or referral to cessation services.^34^

The tobacco industry has manipulated cigarettes to be as addictive as possible and exploited rural Appalachian cultural values to drive tobacco use. The industry has spent decades manipulating nicotine concentration (total nicotine available in a product) and nicotine form (fraction of free-base nicotine [FBN], which is harsher but more readily absorbed^35^) in cigarettes and oral tobacco products. ^11,35,36^ These manipulations created a range of products that appeal to new users (low nicotine concentration and low FBN), cultivate and maintain nicotine dependence (greater nicotine concentration and FBN), and increase the difficulty of cessation among established users (highest nicotine concentration and FBN).^11,12^ In addition to these product-level factors, which affect all people who use tobacco, the industry has targeted rural Appalachia specifically with advertising campaigns that reinforce rural Appalachian cultural values of masculinity, individuality, and ruggedness.^6,13^

Oral nicotine pouches (ONPs) present a new opportunity to reduce cancer risk among Appalachian adults who smoke. Small white pouches that contain nicotine but no tobacco leaf, ONPs entered the US market as a consumer product in 2016^14,37,38^ and sales quickly skyrocketed: 80% FBN, respectively) than Swedish snus.^49,50^ In other words, US snus appears to be designed for new tobacco users with low nicotine dependence, whereas Swedish snus is designed for established tobacco users with high nicotine dependence. As described, the availability of ONPs with varied nicotine concentrations and forms indicate that, unlike snus, there are varieties that could appeal to new and established tobacco users alike. Our preliminary data support this assertion, with rural Appalachian smokers describing ONPs as being more appealing than NRT and more socially acceptable than smoking.

Should this switching study be conducted in a sample of SLT users rather than smokers? The limited surveillance data available on ONP use among adults identified that SLT users are the group with the highest rates of ONP uptake. ^39,40^ Given similarities between the products in their use topography and nicotine delivery,^51– 53^ as well as sales data suggesting that ONPs are cutting into moist snuff’s market share,^37^ this is unsurprising. For these reasons, we strongly considered conducting this study in a sample of moist snuff users (the most popular type of SLT) rather than cigarette smokers, as it was not financially feasible to study both groups. However, we decided to study cigarette smokers for the following reasons: (1) Cigarette smoking is the more prevalent behavior, both in Appalachia and nationally. In the US overall, the prevalence of SLT use is 2.1% (4.2% among men and 0.2% among women).^54^ In Appalachia, the prevalence of SLT use varies but does not exceed 8-9%.^54^ Both of these estimates are markedly lower than for cigarette smoking (i.e., 11.5% nationally and >30% in some rural Appalachian counties). ^4,5^ (2) Cigarette smoking exposes uses to more carcinogens. Moist snuff exposes users to several harmful and potentially harmful constituents that increase risk of cancer.^55,56^ However, the combustion that occurs during cigarette smoking exposes the user (and via secondhand smoke, others nearby) to a substantially greater burden of carcinogens and other toxicants.^57^ In recognition of these differences in risk, the FDA authorized one popular brand of moist snuff as a Modified Risk Tobacco Product in March 2023.^58^ For these reasons, we expect that the public health benefits of smokers switching to ONPs would be greater than for SLT users switching to ONPs.

In summary, ONPs are a promising but unexplored approach to reduce smoking-related harms in rural Appalachia. In the past year, the prevalence of cigarette smoking among adults in the US dropped another percentage point, from 12.5% to 11.5%.5 Less than 10 years ago, the prevalence of smoking was over 20%.5 This striking public health success has not been realized across all areas of the US, however. In several rural Appalachian counties, the prevalence of smoking among adults exceeds 30%.4 Assessments of ONPs’ chemical characteristics suggest that their wide range in nicotine concentrations and FBN fractions will appeal to cigarette smokers, and further, that they will expose users to far fewer toxicants than cigarettes or other forms of oral tobacco. As described below, we have also found that ONPs appeal to rural Appalachian smokers specifically, relieve nicotine withdrawal symptoms, and have been used by rural Appalachian smokers to cut back on their smoking. By directly comparing ONPs to combination NRT on smoking outcomes in a large sample of rural Appalachian smokers, our results will inform public health practitioners, regulators, and healthcare providers about the role that ONPs could play in tobacco harm reduction. If we find that ONPs increase short-term and sustained switching success, these findings support their promotion to smokers looking to stop smoking. If we find that ONPs perform worse or no different than NRT, these findings will indicate that smokers should be directed toward NRT or other methods for smoking cessation, and that regulations to maximize the appeal of ONPs among smokers and limit their appeal among people who do not use tobacco might be needed to protect public health.

**Intervention Impact**

To our knowledge, this is the first study investigating whether cigarette smokers can successfully switch to ONPs. Excluding our preliminary studies, we are aware of no independent research reporting on ONPs’ acute (e.g., plasma nicotine delivery, withdrawal relief) or longer-term (e.g., cigarette abstinence) use effects among smokers. The tobacco industry has funded the published studies of ONPs’ appeal among smokers,^51–53,59^ raising concerns about the objectivity of the evidence base, as well as the generalizability of results beyond the narrow goals of each study. We will provide the first independent investigation of whether ONPs can be used to reduce cigarette smoking and promote cigarette abstinence among smokers.

Conducting this study remotely in rural Appalachia will advance knowledge about the effects of ONPs on smoking outcomes in a region where traditional approaches to tobacco control have been less effective. Smoking cessation success in rural Appalachia is lagging behind the rest of the US. With cultural, healthcare system, and industry-related factors bolstering tobacco use in Appalachia, we are proposing an innovative approach to reduce the burden of cigarette smoking across an entire region. We expect that participants in the ONP arm will experience greater short-term and sustained switching success than participants in the NRT arm because ONPs are marketed and regulated as tobacco products (rather than drugs), are easier to access, and are designed to be appealing to smokers (e.g., higher nicotine concentrations). If these assumptions are proven correct, their promotion to and adoption by smokers could reduce lung and oral cancer disparities that have thus far shown no signs of narrowing.^1^

Our use of a strong, FDA-approved, active control (combination NRT) will provide clarity to public health officials, regulators, and healthcare providers about whether ONPs could be useful for smokers who have been unable to quit smoking using NRT. A human laboratory comparison of different forms of NRT and ONPs in a sample of smokers found that NRT lozenges and ONPs (both 4mg nicotine concentration) had similar plasma nicotine delivery, but ONPs were rated as more appealing.^59^ In addition to nicotine lozenges, participants in our control arm will also receive the transdermal patch, as combination NRT is more effective for smoking cessation than use of a single form of NRT.^60^ With ONPs rapidly increasing in popularity^38^ and rigorous population-level surveillance of their use lacking, conducting this first, crucial assessment of whether ONPs can out-perform combination NRT for smoking abstinence or reduction will elucidate whether they could offer any public health benefit.

**Preliminary Research**

Our preliminary studies demonstrate our expertise in conducting research with oral nicotine products, including remote clinical trials and naturalistic switching studies. Our preliminary research also highlights our expertise conducting research with rural Appalachian populations.

Qualitative assessment of ONPs among rural Appalachian adults who smoke. Dr. Keller-Hamilton conducted focus groups assessing perceptions of ONPs among adult smokers living in Appalachia. Participants perceived ONPs to be lower risk than cigarettes but to be similarly addictive. Participants also noted that ONPs seemed to be “cleaner,” more discreet, and more socially acceptable. Conclusions: rural Appalachian adults who smoke were open to using ONPs and held perceptions that might support switching.

Abuse liability assessment of ONPs among rural Appalachian adults who smoke. Drs. Keller-Hamilton and Wagener collaborated on a pilot randomized cross-over study assessing nicotine pharmacokinetics and subjective effects of ONPs with different nicotine concentrations. In 3 separate visits, N=30 adult smokers from Ohio Appalachia used a 3mg nicotine concentration ONP, 6mg nicotine concentration ONP, or a usual brand cigarette following a standardized use protocol. Participants reported that ONPs were moderately appealing and noted similar overall withdrawal relief across products. Plasma nicotine peaked highest and earliest when smoking a cigarette but using the 6mg ONP resulted in greater plasma nicotine levels than the cigarette from 30-60 minutes of follow-up. Conclusions: (1) The higher nicotine concentrations available in ONPs (than in NRT) support greater plasma nicotine delivery. (2) ONPs relieve withdrawal symptoms and are moderately appealing to rural Appalachian adults who smoke.

Follow-up survey among adult smokers who participated in an acute ONP use clinical trial. After completing the trial described above, several participants contacted us to thank us for introducing them to ONPs and anecdotally shared that they cut down on smoking using ONPs. In response, we developed a 6-month follow-up survey to ask all participants about their tobacco use and perceptions of ONPs. Although cessation was never a goal of the acute use study, 23% of participants who were daily smokers at baseline reported that they now smoke “rarely” or “not at all.” In open-ended responses, participants who reduced or quit smoking noted that ONPs were “stronger” and more enjoyable than they expected and that they were “a helpful tool” for quitting smoking. Conclusion: Without a planned switching outcome, a substantial proportion of rural Appalachian participants switched from cigarettes to ONPs on their own following short-term exposure to ONPs.

Large, nationwide, remote trials of alternative nicotine products. We have significant experience conducting large, nationwide, trials of alternative nicotine products. Co-I Carpenter has led 5 state- or nationwide remote trials of NRT (Ns=616, 849),^61,62^ one nationwide, remote trial of snus (N=1236),^63,64^ and one remote trial of electronic cigarettes (R01CA210625). MPI Wagener is a Co-I on the electronic cigarette trial. MPI Wagener (Dr. Carpenter is a Co-I) also recently completed accrual for a remote, randomized trial (U01DA045537) comparing electronic cigarettes vs. NRT on smoking outcomes among recent tobacco Quitline treatment failures from two states (Oklahoma and South Carolina). This study recruited 350 participants within 18 months with 80% retention. Each of these studies demonstrates our ability to recruit large numbers of smokers, distribute products efficiently via mail, and complete follow-up assessments with strong retention.

Studies collecting data via smartphone and biomarkers remotely. Our team has considerable remote data collection experience using smartphones to complete follow-up assessments (11 studies, including one among rural Appalachian smokers) and Bedfont’s iCO device to collect exhaled carbon monoxide (CO) remotely (4 studies; e.g., U01DA045537). Importantly, these studies have helped us improve upon our participant instructions and troubleshooting procedures to maximize adherence. For example, we significantly improved adherence to the iCO device from 61% to 87% by simply modeling its use via video calls/conference (e.g., Zoom, FaceTime), in addition to written and pictorial instructions.

# **2. Objectives**

**Primary Objectives and Outcome Measures**

This study will be carried out in a group of adults (≥21 years old) cigarette smokers (n=1000) from rural Appalachian regions of the United States according to the following aims.

**Aim 1:** Evaluate short-term changes in cigarette smoking patterns, including switching, abstinence from cigarettes, and frequency of smoking between ONPs and NRT. At the conclusion of the Switch Phase, the highest rates of (H1a) complete switching (cigarette abstinence + study product use) and (H1b) abstinence from cigarettes will occur in the ONP group. H1c: ONP participants will smoke the fewest cigarettes per day.

**Aim 2:** Compare product appeal, craving, withdrawal relief, and perceived cigarette dependence between ONPs and NRT. Participants randomized to ONPs will report (H2a) greater product appeal and (H2b) greater reduction in craving and withdrawal symptoms than participants randomized to NRT. H2c: Among complete switchers, ONP participants will report greater reductions in cigarette dependence than NRT participants.

**Aim 3:** Examine sustained changes in tobacco use behaviors, including abstinence from cigarettes, purchase of ONPs/NRT, and continued use of ONPs/NRT. During the Observation Phase, ONP participants will (H3a) have higher rates of abstinence from cigarettes, (H3b) use their study product for a greater number of days, and (H3c) be more likely to purchase their study product than participants in the NRT group. With stubbornly high rates of smoking—and consequently lung and oral cancers—existing approaches have been inadequate and cannot be the sole means to reduce smoking in rural Appalachia. We propose to evaluate the effectiveness of a potentially reduced harm tobacco product, ONPs, which has significant appeal to rural Appalachian smokers. With expertise conducting remote, large-scale switching trials, our team is uniquely suited to conduct this investigation. This urgently needed and innovative study will be critical for public health officials, regulators, and healthcare providers working to reduce cancer disparities in rural Appalachia.

# **3. Study Design & Procedures**

|  | Y | N | Comments |
| --- | --- | --- | --- |
| Is this a multi-site study? |  |  | If yes, complete the Multi-Site Research appendix. |

While there is a Co-Investigator from the Medical University of South Carolina (MUSC), Ohio State is leading all research activities of this fully remote study, including recruitment and study implementation. There will be regular team meetings where the MUSC Co-I will be updated and able to provide input.

**Research Design**

We will use a 6-month, two-arm, remote randomized switching trial of 1,000 smokers living in rural Appalachia. After randomization to the ONP or NRT (patch and lozenge) arm, during a 2-week Sampling Phase, participants will be sent ONPs or NRT in varied flavors and nicotine concentrations. Participants will sample and familiarize themselves with use of their study product and will select a nicotine concentration and flavor to use for the switch phase. During the 12-week Switch Phase, participants will attempt to stop smoking cigarettes and entirely switch to their study ONP or NRT. During the 14-week Observation Phase, participants will no longer receive study products but will be followed to evaluate longer-term switching outcomes. Throughout the trial, self-reported changes in cigarette smoking via online surveys will be confirmed using remote carbon monoxide assessment.

**Design Overview**


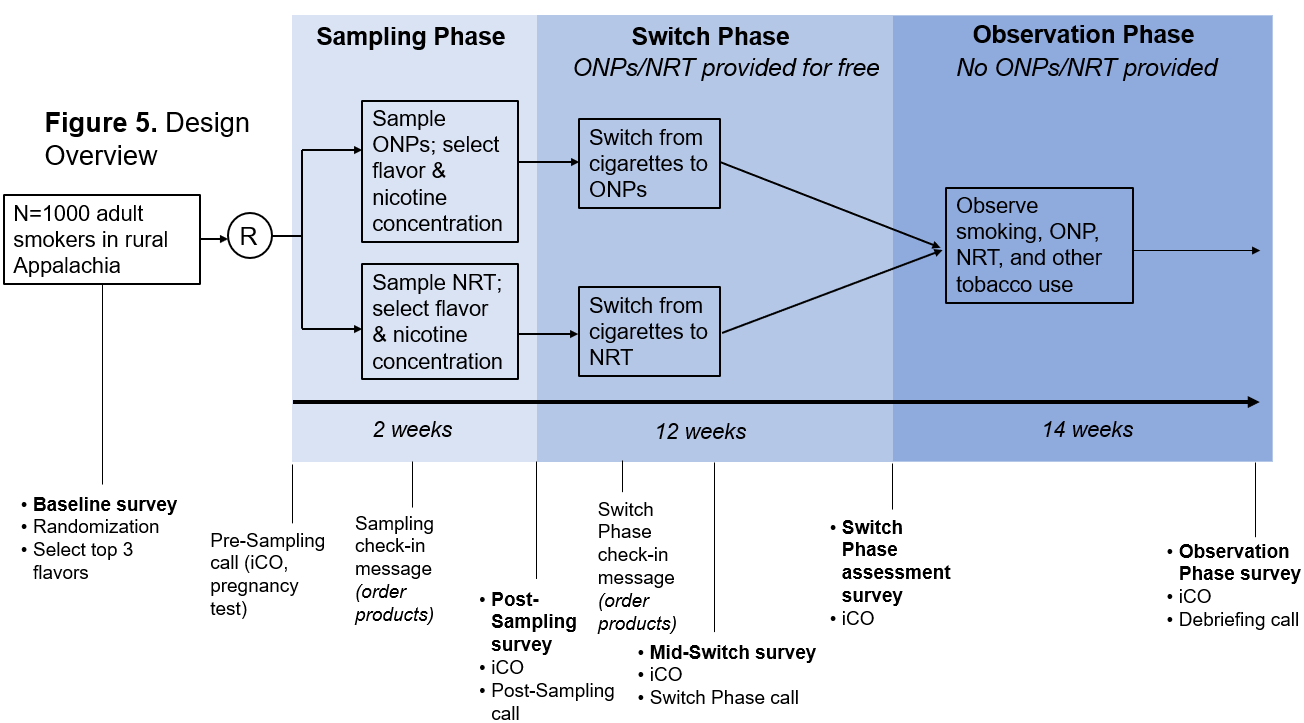


**Detailed Study Procedures**

|  | Y | N | Comments |
| --- | --- | --- | --- |
| Are you requesting expedited IRB review?  Note: The Federal Regulations establish two main criteria for an expedited review:   1. The research may not involve more than "minimal risk" (refer to appendix). 2. The entire research project must be consistent with one or more of the federally defined categories (refer to appendix). |  |  | If yes, complete the Expedited Review appendix. |
| Does the research involve genetic testing? |  |  | If yes, complete the Genetic Testing appendix. |

## Initial Screening

Interested adults will be directed to an online REDCap screening questionnaire for determination of initial eligibility ***(Online Screener)***, where they will see a script that briefly explains the study.

Orientation & Consent

Participants who meet the initial screening criteria will be scheduled for an Orientation Video Call (e.g., Zoom or Teams, per the participant's preference) ***(Schedule Recruit Orient).*** Participants will be sent a reminder for their scheduled Orientation ***(ZOOM Call Reminder).*** If the participant prefers a phone call or if video conferencing is not a good option, the Orientation will take place via phone call. During Orientation, eligibility will be confirmed ***(Verbal Screener)*** and additional study details will be provided ***(Recruit Screen Phone Script).*** If the Orientation is scheduled for Zoom, they will receive Zoom instructions ***(Zoom Instructions Reminder Email).***

Upon confirmation of eligibility and willingness to participate, participants will receive an oral and written explanation of the study. Study staff will review the consent document with participants and answer any questions the participants may have. Participants will demonstrate that they understand key aspects of the study by verbally answering questions from study staff about participation (e.g., “Can you tell me what the risks of participation are?”). The voluntary nature of the study and the participant’s right to withdraw at any time will be stressed during the consent process.

Participants will electronically sign the consent form only after both the participant and the study staff member are confident that the participant understands their participation and the risks associated with participating. The informed consent agreement will be collected by IRB-approved study personnel. Consent signatures will be collected electronically via REDCap ***(Consent Form)*** and stored electronically in the secure REDCap database***.***A copy of the informed consent document will be provided to the participant electronically ***(Copy of Consent Message)*** and a blank hard copy will be included in the study kit. All participants will provide their consent before any study procedures are performed. After consent, with the help of study staff, participants will sign their W-9 (or OSU supplier/payee setup form).

Baseline Phase

At the conclusion of the Orientation Call, participants will be emailed or texted a link to the baseline questionnaire ***(Survey Message, Baseline Survey).*** The baseline survey will ask which flavors the participant would like to sample. Participants will be asked about both NRT and ONPs since randomization will not have occurred yet. Participants will be asked to complete the baseline survey in 3 days. Study staff will call, email or text (any combination based on participant preferences) participants up to 10 times over the course of two weeks to remind them to complete the survey. ***(Survey Reminder).***

Following completion of the baseline survey, participants will be randomized to one of two arms for a period of 14 weeks (the Sampling and Switch Phases): 1) ONPs or 2) combination NRT. Randomization will occur using a stratified block-randomization procedure with small, random sized-blocks. Randomization will be stratified by self-reported cigarette dependence, sex, and ONP use history (ever vs. never). We will use FTND<5 vs FTND>=5 for the cigarette dependence portion of the randomization.

Study Kits/Products: Following randomization, participants will be mailed their study kits. Study kits will be tailored to each study group. Study kits will include a Welcome Card ***(Welcome Card),*** welcome/thank-you letter ***(Welcome Letter),*** copy of the consent form, ***(NRT / ONP Fact Sheet)****,* study product guidelines ***(NRT / ONP Guidelines)****,* study product troubleshooting tips ***(NRT / ONP Tips),*** nicotine overdose fact sheet ***(Nicotine Overdose Fact Sheet)*,** participant roadmap outlining their involvement in the study ***(Participant Roadmap),*** individual carbon monoxide (iCO) device and instructions ***(iCOquit Instructions),*** pregnancy test for those who can become pregnant, and study product to sample. The study kit will also include the participant’s ClinCard, and information on how to use the ClinCard ***(ClinCard Info Sheet, ClinCard FAQs)*** and information about the referral program ***(Referral Letter, Referral Flyer).***

The ONP Group will receive three flavors based on what they chose on the baseline survey. They will receive 3 mg and 6 mg concentrations in each flavor.

The NRT group will receive 14 mg and 21 mg patches, and they will receive one to three flavors of lozenges based on what they chose on the baseline survey. They will receive 2 mg and 4 mg concentrations in each flavor.

All products will be given to participants in their original packaging and at no cost. The packaging will clearly be labelled “For Research Purposes Only.” We will supplement product instructions with additional information on storing products away from children and pets. Although accidental ingestion is very unlikely and has not occurred in our team’s previous studies, all participants will be provided the national poison control telephone line as well as a “tip sheet” on recognizing signs of nicotine overdose. Participants will be instructed to not begin using the products until after the Pre-Sampling Call.

## Sampling Phase

The goal of the two-week Sampling Phase is for participants to become familiar with the study product and choose the nicotine concentration and flavor(s) they will use to attempt to switch to during the first part of the Switch Phase. Additionally, as our prior research demonstrates product sampling is particularly important in supporting smoking cessation success among rural smokers,^70^ the Sampling Phase is intended to strengthen switching outcomes in the Switch and Observation Phases.

Study Products provided during the Sampling Phase (sent to participants in their study kits based on their randomization) include Zyn brand oral nicotine pouches (ONPs) and Nicotine Replacement Therapy (NRT) lozenges and patches.

Oral Nicotine Pouch (ONP) group participants will receive ONP products in up to three flavors (selected via their baseline questionnaire) for the 2-week Sampling Phase: 3mg and 6mg nicotine concentration in each chosen flavor. We will instruct participants to start with lower nicotine concentration pouches and to only escalate to higher nicotine concentration pouches if they are not experiencing adverse effects (e.g., dizziness, nausea). Participants who wish to try the higher nicotine concentration pouches will also be encouraged to try them first for shorter time periods (e.g., 10 minutes) before committing to a full 30- to 60-minute use session.

Nicotine Replacement Therapy (NRT) group participants will receive NRT patches and lozenges. NRT lozenges were selected as an active control condition because they are FDA-approved, available for purchase without a prescription, and have demonstrated efficacy in helping smokers quit.^60^ We will include the initial 2-week provision of 2mg and 4mg lozenges and 14mg and 21mg patches (Rugby brand) for trial during the Sampling Phase. As with ONP instructions, participants will be instructed to start with the lowest nicotine dosages of NRT and to only escalate to higher nicotine concentrations (for shorter time periods to start) if they do not experience adverse effects.

The Sampling Phase will include two calls with study staff, a brief messaging contact halfway through and a self-administered survey, with iCO measurement, at the end of the sampling phase.

Sampling Phase: Pre-Sampling Zoom/Call

The Pre-sampling Zoom or phone call will be scheduled for when participants have received their study kits. Reminders about the call will be sent 1-2 days before the scheduled Pre-Sampling Call ***(PreSamp Call Reminder Text, Zoom Instructions Reminder Email).*** The Zoom/call will include study product education, use instructions, and troubleshooting tips. Participants will also complete their first iCO measurement during the pre-sampling call. This measurement will confirm that they are smoker. If the measurement is ≥7 ppm, they will be considered a smoker. If the measurement is less than 7 ppm, they will not be able to participate. During this call, participants will also verbally confirm that they are not pregnant.

Participants will set an agreed upon switch date approximately 2 weeks from the pre-sampling call and will be asked to immediately start using their sample products ad libitum to become familiar with their use. Participants will also be informed that they will receive two additional product shipments, one before their switch day and another ~6 weeks after their switch day. Participants will be asked prior to future shipments what flavors and nicotine concentrations they would prefer to be sent. ***(Pre-sampling Call Script).***

Sampling Phase: Product Sample Survey

The Sampling Phase check-in message/questions will be automatically sent via REDCap (text or email) 6-7 days after the Pre-Sampling Call. The questions will ask participants to choose study product nicotine concentration(s) and flavor(s) for the first ~6 weeks of the Switch Phase. ***(Product Sample Survey)*** If the questions are not completed in 24 hours, the study team will follow-up with a phone call and text and/or email ***(Survey reminder).*** Study staff will call, email or text (any combination based on participant preferences) participants up to 10 times to remind them to complete the survey if they have not completed it within two weeks. The switch phase may be delayed if the participant has a delayed response to the survey.

Sampling Phase: Post-sampling Survey

The Post-sampling Survey will assess participants’ confidence in switching to their study product, as well as their experiences and extent of sampling during the Sampling Phase ***(Survey Message, Post-Samp Survey****)*. The post-sampling survey will be sent on day 10 after the sampling phase starts. The survey will be completed prior to the Post-Sampling Call. Study staff will call, email, or text (any combination based on participant preferences) participants up to 10 times to remind them to complete the survey if they have not completed it within two weeks. ***(Survey reminder).***

Sampling Phase: Post-sampling Call

The Post-sampling Call will be one to two days before the participant’s switch date. Study staff will confirm receipt of additional products, remind participants of their switch date and that upon waking the morning of their Switch Date they should not smoke cigarettes. They will be instructed to stop smoking and continue with their allocated study product (ONP or NRT) for 12 weeks. Staff will also answer any product use questions and provide troubleshooting as needed. ***(Post-Samp Call Script)*** If study staff are unable to connect with the participant, the participant will be emailed or texted the information ***(Post-Samp Email).***

## Switch Phase

The Switch Phase will start on a participant’s “switch date” and will last for 12 weeks. Study participants will receive a reminder text the day before and on the day of their Switch Day ***(SD Reminder Text).*** The goal of the Switch Phase is to identify whether there are differences in cigarette smoking patterns (i.e., switching to the study product, abstinence from cigarettes, and frequency of smoking) between the ONP and NRT groups. We will also assess potential differences in subjective measures of product appeal, craving and withdrawal relief, and changes in perceived cigarette dependence between groups.

Switch Phase: Product Sample Survey

The Switch Phase product sample survey will be automatically sent via REDCap 4 weeks after switch day. ***(Product Sample Survey).*** Participants will be asked to complete questions on their study product preferences for nicotine concentration and flavor for their next (and last) product shipment. Study staff will call, email or text (any combination based on participant preferences) participants up to 10 times to remind them to complete the survey if they have not completed it within two weeks. ***(Survey Reminder)***

Switch Phase: Mid-Switch Survey

The Mid-Switch Survey will be self-administered via REDCap 6 weeks after the switch date to assess product appeal, craving and withdrawal, perceived cigarette dependence, and smoking and study product use behaviors; this survey will also include an iCO assessment. ***(Survey Message, Mid-Switch Survey)*** Study staff will call, email or text (any combination based on participant preferences) participants up to 10 times to remind them to complete the survey if they have not completed it within two weeks. ***(Survey reminder).***

Switch Phase: Mid-Switch Phone Call

The Switch Phase Mid-Switch Phone Call will occur approximately 6 weeks after the switch date, after participants have received their product shipment. This call will provide an opportunity to check-in with participants to confirm receipt of their final shipment and answer any product- or study-related questions. Participants will also be reminded about completing the Switch Phase assessment survey that occurs at the end of Switch Phase (12-weeks after switch date). ***(Mid-Switch Phone Call Script)*** If study staff are unable to connect with the participant, the participant will be emailed or texted the information ***(Mid-Switch Email).***

Switch Phase: Post-Switch Survey

The Post Switch survey will be self-administered via REDCap 12 weeks after the switch date to measure product appeal, craving and withdrawal, perceived cigarette dependence, and changes in smoking patterns (e.g., abstinence from cigarettes, frequency of smoking, use of the study product). The Switch Phase assessment survey will also measure iCO. ***(Survey Message, Post-Switch Survey*)** Study staff will call, email or text (any combination based on participant preferences) participants up to 10 times to remind them to complete the survey if they have not completed it within two weeks. ***(Survey reminder).***

## Observation Phase

The Observation Phase will begin immediately after the Switching Phase and will last for 14 weeks. The goal of the Observation Phase is to assess changes in smoking and sustained use of study products when they are no longer provided for free. Secondary goals will include assessing changes in nicotine concentrations, flavors, and brands used, as well as uptake of the other nicotine/tobacco products. This phase will help us to continue to track any adverse events that may occur following use of the study products.

Observation Phase: Survey

Participants will complete a survey at the end of the Observation Phase. ***(Survey Message, Observation Survey)*** The Observation Phase Survey will measure smoking and product use outcomes as described in the previous paragraph, including an iCO assessment. It will be delivered via REDCap and participants will receive several automated notifications to complete the survey. Study staff will also call, email, or text (any combination based on participant preferences) participants up to 10 times to remind them to complete the survey if they have not completed it within two weeks ***(Survey reminder).***

Observation Phase: Phone Call

After the survey is submitted, participants will receive a debriefing call. ***(Debrief Call Schedule Reminder)*** During the Debriefing Call, participants who report continued cigarette smoking will be strongly advised to quit smoking and will be emailed cessation resources ***(Cessation Resources).*** Those who have completely switched to ONPs or NRT will be encouraged to continue not smoking cigarettes and will be emailed cessation resources to assist with quitting all nicotine. ***(Debrief Phone Call Script)*** If we are unable to reach the participant by phone, they will receive a debrief email ***(Debrief Email).***

## Data Collection and Management

Across all aims, data will be collected and stored electronically in the secure database, REDCap. Data will only be accessible to approved study personnel and will be secured through multiple firewalls. Subjects’ first and last names will be deidentified and not linked with the data. The consent forms with first and last names will be stored electronically in REDCap but will not be downloaded with any study data. Survey data will be collected via REDCap on participants electronic devices.

Leveraging REDCap for survey links will reduce the possibility of study staff errors.

## Procedure Manual

Study procedures, collecting iCO measurements, completing and logging phone calls, assembling and shipping kits, gift card administration, and other procedures will be thoroughly explained in the study procedure manual.

### Obtain exhaled iCO

iCO measurements will be collected according to the participant iCO Instructions at timepoints throughout the Baseline, Sampling, Switching, and Observation phases.

Bedfont iCO Smokerlyzer monitor (Bluetooth version) will measure exhaled CO levels and

biochemically confirm smoking status over the course of the study. Participants will be prompted to connect the iCO device to their phone following completion of each assessment and provided step-by-step directions on how to complete the test. Results will be date and time stamped and saved. Our CO criteria for smoking abstinence is consistent with numerous other studies using cutoffs of ≤ 6 ppm.^87–92^ As ONPs and NRT do not produce CO, exhaled CO is a valid indicator of smoking status and compares favorably with cotinine and other biochemical measures that have longer detection windows.^93–96^ Self-reports of abstinence combined with CO levels suggestive of recent abstinence provide an accurate, immediate, and practical measure of abstinence from cigarettes. The manufacturer indicates that the iCO is valid for approximately 200 CO tests and has a sensor sensitivity of 1 ppm.^97^ Our protocol will require no more than 5 CO testing sessions—well within the defined valid use range.

### Pregnancy Test

All subjects capable of becoming pregnant will be sent a pregnancy test in the study kit. Each participant will need to verbally confirm that they are not pregnant during the pre-sampling call based on the results of the pregnancy test. If a participant is unable to confirm that they are not pregnant or if they are pregnant, they will not be able to continue with the study.

## Randomization, Blinding, and Unblinding

All randomization procedures will be stratified to ensure balance within groups. After baseline, participants will be randomized within the secure database, REDCap. Randomization will occur using a stratified block-randomization procedure with small, random sized-blocks. Randomization will be stratified by self-reported cigarette dependence, sex, and ONP use history (ever vs. never). Randomization will be counterbalanced and will not be changed once it is complete.

Participants are not blinded to their study product(s) and are instead active participants in trialing their products.

## Subject Completion, Withdrawal, and Early Termination

Participants will be invited to complete a 29-week, fully-remote study involving baseline, sampling, switching, and observational phases. The participant timeline will be complete once the Observation Phase Survey and Debriefing Calls are complete. Participants have the option to withdraw at any point by expressing their desire to withdraw to study staff orally or in writing. Study staff will complete a withdrawal form to document this event. If at any point a participant becomes unable to complete study tasks per protocol (e.g., inconsistent responses reported on questionnaires; inability to provide iCO or survey responses; injury, illness, or medications that impair ability to accurately complete study measures; desire to quit tobacco use; or inappropriate behavior toward study staff), Dr. Keller-Hamilton reserves the right terminate a subject’s participation in the study based on their safety and ability to complete the protocol.

### Participant Timeline

Participants will enroll and participate fully remotely from an electronic device. The participant timeline includes a Baseline Phase (up to 1 week), Sampling Phase (2 weeks), Switching Phase (12 weeks), and Observation Phase (14 weeks) for a total of up to 29 weeks.

Participant time commitment:

- Verbal Screening/Consent/Orientation: 25 minutes
- Baseline Survey: 25 minutes
- Pre-sampling call/Review of study kit: 30 minutes
- Product sample survey, first 6 weeks: 5 minutes
- Post-sampling survey: 25 minutes
- Post-sampling phone call: 10 minutes
- Product sample survey, second 6 weeks: 5 minutes
- Mid-switch survey: 25 minutes
- Mid-switch phone call: 10 minutes
- Post-switch survey: 25 minutes
- Observation survey: 25 minutes
- Debrief phone call: 10 minutes

Total time commitment: 220 minutes (3 hours, 40 minutes)

### Duration of Study

We anticipate the clinical trial to take 5 years to complete. The groundwork for data collection will take place in Y1, recruitment and enrollment activities will take place in years 1, 2, 3, and 4, and data analysis will be the focus in year 5.

**Alternatives to Participation**

They may choose not to participate without penalty or loss of benefits to which they are otherwise entitled.

# **4. Institutional Approvals**

| Check all institutional approvals that apply to research being conducted at Ohio State. |
| --- |
| Comprehensive Cancer Center (CCC) Clinical Scientific Review Committee (CSRC): Approval or exemption required prior to IRB review for all cancer-related research.  Provide a copy of the approval letter on the Other Files page of the smart form. |
| Institutional Biosafety Committee (IBC):  Approval required prior to IRB review for research involving biohazards (recombinant DNA, infectious or select agents, viruses, toxins), gene transfer, or xenotransplantation.  Provide a copy of the approval letter on the Other Files page of the smart form. |
| Human Subject Radiation Committee (HSRC):  Approval required for research involving radiologic procedures for research purposes (e.g., non-clinical care X-rays, DEXA or CT scans, nuclear medicine procedures).  Complete the Radiation appendix and provide a copy of the HSRC approval letter if available. |

# **5. Participant Population**

| Specify the participant population(s). Check all participant groups that apply. For any population other than adults, complete the applicable appendix. |
| --- |
| Adults with impaired decision-making capacity  Children  Neonates of uncertain viability  Nonviable neonates  Non-English speaking individuals  Pregnant women/fetuses (only if pregnant women will be intentionally recruited and/or studied)  Prisoners |

We will recruit 1000 participants from the 180 rural counties in the Appalachian region. In alignment with the Appalachian Regional Commission,^8^ we will use Urban Influence Codes (UICs) from the United States Department of Agriculture and Office of Management and Budget to identify rural counties; counties with a UIC > 6 will be eligible for inclusion.

## Inclusion/Exclusion Criteria

| ***Inclusion and Exclusion Criteria*** | |
| --- | --- |
| **Inclusion** | **Exclusion** |
| Read and speak English | Report currently using smoking cessation medications, NRT, or actively seeking treatment for smoking cessation |
| 21 years or older | Use of ONPs in past 3 months |
| Smoke ≥5 cigarettes per day for the past year | Use of Nicotine Replacement Therapies in past 3 months |
| Willing to use ONPs or NRT | Use of other tobacco products >10 days/month |
| Live in rural Appalachian county | Unstable or significant medical condition |
| Have a smartphone and/or willing to use a smartphone for study purposes (smartphones may be provided to participants for study purposes only); willing to download a free app for the study | Unstable or significant psychiatric conditions (past and stable conditions will be allowed) |
| If capable of becoming pregnant, verbal confirmation of not being pregnant based on urine pregnancy test results | History of cardiac event or distress within the past 3 months |
| Willing to blow air into a handheld study device | Currently pregnant, planning to become pregnant within 6 months, or breastfeeding |
|  | Live in same household as another study participant. |

Note: Only participants who complete the baseline assessment, verify themselves as a smoker at the pre-sampling call time-point via iCO ≥7, and confirm they are not pregnant (if capable) will be included as part of the intent-to-treat sample. If the participant’s iCO reading is below 7, study staff will call the participant to ask when they last smoked/when they took the iCO test. The participant may be asked to retake the test at a more typical time if the initial test was unusual, e.g., before the first cigarette of the day.

We are excluding people under the age of 21 due to tobacco and nicotine use being illegal for this age group. We are also excluding pregnant or breastfeeding individuals or those who are not able to produce a negative pregnancy test due to NRT and ONP not being recommended for use during pregnancy. We are excluding participants who do not speak and read English as our study forms are all in English.

#### Inclusion of Women and Minorities

Women will be included in the project. Currently, women represent approximately 50% of smokers. Our lab has traditionally recruited approximately 50% of its sample as women when recruiting adult smokers, and we expect that to be the case here. If the share of either sex in our sample exceeds 55% during accrual, we will pause recruitment advertisements to the over-represented sex to improve balance within our sample.

Participants from racial and ethnic minority groups will be included. Participants will be recruited from the 180 rural Appalachian counties in the US. According to the 2020 US Census, the racial and ethnic distribution in Appalachia is 80.3% white non-Hispanic, 10.0% Black non-Hispanic, 5.6% Hispanic (any race), and 4.1% other race, non-Hispanic.^8^ We will target enrolling a sample that closely matches the racial and ethnic distribution of the Appalachian region. We will continuously monitor enrollment to ensure we are meeting recruitment goals to avoid under-recruiting minority race and ethnicity participants. If the targeted enrollment for minorities is not met because they do not respond to the advertisements, we will make special efforts to increase their participation by advertising in community newspapers, community centers, and working with the OSU Comprehensive Cancer Center’s Center for Cancer Health Equity (<https://cancer.osu.edu/our-impact/community-outreach-and-engagement/center-for-cancer-health-equity>). We will also pause recruitment advertisements to over-represented groups as needed to improve balance in our sample. We will require that no more than 82% of our sample is white non-Hispanic.

**Number of Participants**

We expect up to 1,000 participants to enroll in this study. Each subject must meet I/E criteria.

Assuming 80% retention at the end of the 12-week Switch Phase, a total sample of 800 participants (400 per arm), will provide greater than 95% power to detect a difference in smoking abstinence between the NRT and ONP arms for a two-sided, a=0.05 level chi-squared test. Further assuming 70% retention at the end of the 14-week Observation Phase, a sample of 700 (350 per arm) will provide over 90% power to detect significant difference between the ONP and NRT arms.

All study activities will take place remotely using participants’ study devices.

**Participant Identification**

Leveraging various recruitment methods, we will recruit participants from the 180 rural counties in the rural Appalachian region. In alignment with the Appalachian Regional Commission, we will use Urban Influence Codes (UICs) from the United States Department of Agriculture and Office of Management and Budget to identify rural counties; counties with a UIC > 6 will be eligible for inclusion.

We will identify participants through following recruitment methods:

1. Online Advertising
2. Referrals
3. Lung Cancer Screening Activities
4. Community Organizations
5. Marketing Emails
6. Contacting participants from other studies
7. Newspapers
8. Webpage
9. Referrals
10. Respondent Driven Sampling

Online Advertising

We will utilize platforms including but not limited to Meta (ex. Facebook, Instagram), Google (ex. YouTube, ads), online advertising, and TikTok for recruiting. ***(Social Media; BC Ad Copy, Feed, Landing Page, Screening Form, Snapchat, Video 1, Video 2, Video 3, Video 4)***

Lung Cancer Screening Activities

Study information will be shared via lung cancer screening activities, such as lung cancer screening vans visiting areas of Appalachia. ***(Flyer)***

Community Organizations

We will work with local community groups and or organizations, such as community coalitions and local health departments, to promote the study. ***(Flyer) (Ads)***

Marketing Emails
Study staff will work with agencies who are able to promote the study via marketing emails. ***(Flyer) (Ads)***

Contacting participants from other studies

Study staff will contact participants from other studies who have consented to be contacted about additional studies. ***(Recruit Email) (Recruit Text)***

Newspapers

Ads will be utilized in local newspapers (print and online) to promote the study. ***(Ads)***

Webpage

There will be a study webpage ***(Webpage)*** with information, including the link to the online screening survey.

Referrals
Study participants may share study information with others who may be interested. ***(Flyer)***

Respondent Driven Sampling

Subjects will also be recruited through respondent-driven sampling (RDS), which is commonly used in studies involving contact tracing and social networks (e.g., sexual health studies). This sampling method is a type of peer-driven chain-referral sampling (Heckathorn 1997, 2002). Although there are biases associated with chain-referral sampling that can affect the composition of the sample achieved, RDS can control these biases through its methods of data collection and analysis. RDS initial recruiters who are study subjects will recruit future subjects (seeds) who in turn recruit other subjects (seeds). This chain of recruiters and recruits then continues for multiple “waves” of recruitment. Ongoing recruitment is fostered with a dual incentive system: one incentive for participating in the project and another incentive for each person recruited. Recruiters are linked to their seeds by a unique number or code on recruitment coupons/flyers, and they are limited in how many people they can recruit. The identity and status of individuals who may have provided RDS codes/coupons will not be disclosed. The seeds who meet the eligibility can choose to participate in the study or not. Referring individuals to the study is optional. Subjects’ participation in the study will not be affected if they do not invite others or if those they invite do not meet the criteria outlined above. Participants will be provided with referral information in their study kits including their codes ***(Referral Flyer, Referral Letter).*** Participants can share the ***ARISE Referral Image*** with people they believe may be interested and a good fit for the study. We may share the referral image via email or text. We may also share the referral program information via email ***(Referral Email).*** Their referral code will be linked to their record and the payment will be made. Participants can $10 for each 21+ year old smoker they refer. To be eligible for referral compensation, the person referred must complete the online and verbal screeners. Participants can earn up to $50.

We have significant experience conducting large, nationwide, trials of alternative nicotine products. Co-I Carpenter has led 5 state- or nationwide remote trials of NRT (Ns=616, 849), one nationwide, remote trial of snus (N=1236), and one remote trial of electronic cigarettes (R01CA210625). MPI Wagener is a Co-I on the electronic cigarette trial. MPI Wagener (Dr. Carpenter is a Co-I) also recently completed accrual for a remote, randomized trial (U01DA045537) comparing electronic cigarettes vs. NRT on smoking outcomes among recent tobacco Quitline treatment failures from two states (Oklahoma and South Carolina). This study recruited 350 participants within 18 months with 80% retention. Each of these studies demonstrates our ability to recruit large numbers of smokers, distribute products efficiently via mail, and complete follow-up assessments with strong retention.

**Participant Recruitment and Selection**

Recruitment through our recruitment methods requires the individual to complete an online screening survey. It will be up to the person to take the initiative to complete the online screening survey on their own time from their location of choice. Beyond study staff, no one will know who completes the online screener unless the participant chooses to share the information. The screener itself is maintained in a secure database, REDCap, and only study staff will be able to access their responses. Additionally, prospective participants can close out the screener at any time. Moreover, study questions are not designed to be harmful and instead focus on data collection. Recruitment involving former or current study participants who consented to recontact for future studies will also be contacted. We will only contact those who permitted contact on their consent forms through their approved email address or phone number.

Participants who meet the initial screening criteria via the online survey will be scheduled for an Orientation Video Call (e.g., Zoom or Teams, per the participant's preference). During the orientation, eligibility will be verified. A private location will be encouraged for the Orientation Zoom/call.

# **6. Cost to Participants and Incentives to Participate**

**Potential Costs/Reimbursements**

Costs associated with taking part in the study include any cell phone plan or WIFI related costs (needed to complete surveys online and participate in study calls). If any medical issues arise as a result of the study, these would be covered by the participant and not the study.

All nicotine pouch and nicotne replacement therapy costs will be covered by the study.

**Incentives**

Participants will be provided with a ClinCard for incentives. $25 will be loaded to the participant’s ClinCard for each survey/assessment completed and a $25 bonus will be added for completing all 5 surveys/assessments. Participants may also receive $10 for each smoker they refer who completes the online and verbal screenings. They may refer up to 5 smokers. The total incentive is up to $200.

# **7. Compensation for Research-Related Injury**

If a participant suffers an injury from participating in this study, they should notify the researcher or study doctor immediately, who will help determine if they should obtain medical treatment.

The cost for this treatment will be billed to the participant or their medical or hospital insurance. The Ohio State University has no funds set aside for the payment of health care expenses for this study.

# **8. Informed Consent Process**

| Specify the consent process(es) to be used for the study. Check all processes that apply. For any waivers or alterations, complete the applicable appendix. |
| --- |
| Informed Consent with Written Signature (e.g., on paper)  Informed Consent with Electronic Signature  Note: If the method used to obtain electronic signatures does not qualify as a legally valid signature, also check “Waiver of Consent Documentation.” See guidance provided by the [Office of Technology and Digital Innovation](https://u.osu.edu/esigforinformedconsent/).  Waiver of Consent/Parental Permission Documentation  Deception (i.e., procedure in which investigators deliberately mislead participants during research by withholding information or providing false information).  Note: If this is selected, also check “Waiver or Alteration of the Consent Process.”  Waiver or Alteration of the Consent Process  Waiver of the Parental Permission Process |

Upon confirmation of eligibility and willingness to participate, participants will receive an oral and written explanation of the study. Study staff will review the consent document with participants and answer any questions the participants may have. Participants will demonstrate that they understand key aspects of the study by verbally answering questions from study staff about participation (e.g., “Can you tell me what the risks of participation are?”). The voluntary nature of the study and the participant’s right to withdraw at any time will be stressed during the consent process.

Participants will electronically sign the consent form only after both the participant and the study staff member are confident that the participant understands their participation and the risks associated with participating. The informed consent agreement will be collected by IRB-approved study personnel. Consent signatures will be collected electronically via REDCap ***(Consent Form)*** and stored electronically in the secure REDCap database***.***A copy of the informed consent document will be provided to the participant electronically ***(Copy of Consent Message)*** and a blank hard copy will be included in the study kit. All participants will provide their consent before any study procedures are performed.

We aim to minimize coercion or undue influence by:

- Educating the participant as much as possible and providing ample time to ask questions and contemplate prior to completing consent
- Incentive amounts are nominal
- Although we are using addictive products, the products are commercially available, and all available evidence suggests they are much less harmful than cigarettes.

# **9. Privacy of Participants**

Recruitment through our recruitment methods requires the individual to complete an online screening survey. It will be up to the person to take the initiative to complete the online screening survey on their own time. Beyond study staff, no one will know who completes the online screener unless the participant chooses to share the information. The screener itself is maintained in a secure database, REDCap, and only study staff will be able to access their responses. Additionally, prospective participants can close out the screener at any time. Moreover, study questions are not designed to be harmful and instead focus on data collection. Recruitment involving former or current study participants who consented to recontact for future studies will also be contacted. We will only contact those who permitted contact on their consent forms through their approved email address or phone number.

All Zoom/phone calls will be encouraged to take place in a private setting for both the staff member and participant. All surveys are sent to the participant's email and/or texted to the cell phone number provide by the participant.

# **10. Confidentiality and Management of Study Materials**

|  | Y | N | Comments |
| --- | --- | --- | --- |
| Does the research involve obtaining and storing participants’ data and/or biospecimens for future, unspecified, research? |  |  | If yes, complete the Repositories appendix. |

Confidentiality will be maintained by all study staff along with the use of a secure study database. All lab personnel and study staff will complete HIPAA and CITI trainings, human subjects protection training and responsible conduct of research training. Phone scripts will be developed to ensure the subject is in a private environment where they are comfortable sharing personal information during the Orientation Zoom/call. REDCap, will store participant data. A subject ID will be assigned to each participant in order to code study data. Multiple levels of security clearance are required before entering the REDCap system and only approved study personnel will have access to this data.

All data will be collected and stored electronically in the secure database, REDCap. Subjects’ first and last names, phone numbers, and addresses will be coded and not linked with downloadable study results. Data will be stored under a code, not first or last name. Primary research data should be retained for a minimum of five years after final project closeout.

Research will be conducted in accordance with CTR policies and IRB regulations. Questions will be directed to OSU IRB representatives and S/AEs will be reported per IRB policy. All participant data is stored electronically within REDCap with no hardcopy records.

The data quality control process involves randomly sampling completed records each quarter to assess their completeness and accuracy. This ensures that all required fields are filled out properly and that no critical data is missing. Additionally, discrepancies or inconsistencies are flagged for review and correction. The process also includes regular audits to verify the integrity of the data and ensure compliance with study protocols. By systematically monitoring data quality, we can identify potential issues early and maintain the reliability of the study results.

Study procedures, collecting iCO measurements, completing and logging phone calls, assembling and shipping kits, gift card administration, and other procedures will be thoroughly explained in the study procedure manual.

For the iCOquit app, the participant will need to create an account with their email and a password. The app will ask for name and age. There are questions about their smoking habits, but they are optional (cigarettes smoked a day, price per pack, minutes before first cigarette of the day, etc). The parent company, Covita, does not have access to the information/data that is collected by the app.

# **11. HIPAA Research Authorization**

| Is individually identifiable Protected Health Information (PHI) subject to the [HIPAA Privacy Rule](https://go.osu.edu/hipaaprivacysummary) requirements to be accessed, used, or disclosed in the study?  No  Yes |
| --- |
| If PHI is accessed, used, or disclosed, specify how authorization requirements will be met (check all that apply). For any waivers or alterations, complete the applicable appendix. |
| Written Authorization  Partial Waiver (for identification and recruitment purposes only)  Full Waiver (authorization will not be obtained)  Alteration (written authorization will not be obtained or all required elements will not be included) |

# **12. Reasonably Anticipated Benefits**

Participants are not expected to benefit from this project; however, it is possible they may consider reducing their tobacco intake or switching to a less harmful product as a result of their interactions with research staff or the study product(s).

Participants assigned to the Control group will receive combination NRT, the leading forms of pharmacotherapy for smoking cessation, free of charge. Participants assigned to the ONP condition will receive a product that is likely much less harmful than conventional cigarettes to completely switch to for 12 weeks, free of charge. In general, this study will provide a benefit to the public as the findings will be reported to health officials and the US FDA to inform effective tobacco control policies. In an ever-changing marketplace of tobacco and nicotine products, ONPs are non-combustible and tobacco-leaf-free alternatives to cigarettes, although the potential for them to serve as substitutes is largely untested. The current trial will provide information about whether ONPs can be an effective method to reduce the harms of cigarette smoking among rural Appalachian adults who smoke. Participants may indirectly consider quitting tobacco products or choosing a harm reduction product as a result of their involvement with this investigation though this is not a study objective. Through their contributions to science, subjects provide insight into a novel tobacco product that could benefit public health and alleviate the burden of tobacco use.

In general, this study will provide a benefit to the public as the findings will be reported to health officials and the US FDA to inform effective tobacco control policies. In an ever-changing marketplace of tobacco and nicotine products, ONPs are non-combustible and tobacco-leaf-free alternatives to cigarettes, although the potential for them to serve as substitutes is largely untested. The current trial will provide information about whether ONPs can be an effective method to reduce the harms of cigarette smoking among rural Appalachian adults who smoke. Through their contributions to science, subjects provide insight into a novel tobacco product that could benefit public health and alleviate the burden of tobacco use.

# **13. Risks, Harms, & Discomforts**

Every effort will be made to minimize risks to participants. Participation in surveys involves minimal risk. However, there is a possibility of loss of confidentiality or privacy. Additional potential risks include: the risk of smoking cigarettes, using oral nicotine pouches or nicotine replacement products, possible interference with your plans to quit smoking, and the potential for developing side effects from the study products. These side effects may include hiccups, gum irritation, mouth sores, nausea or upset stomach, headache, sore throat, cough strange or vivid dreams, trouble sleeping, dizziness, heartburn, and redness, swelling, burning or irritation at the patch site. Stop using the study product and seek medical care if  you experience irregular heartbeats or palpitations, severe chest pain or tightening, painful mouth sores or blisters that do not resolve, severe skin irritation or discoloration, symptoms of an allergic reaction, or symptoms of nicotine overdose (like extreme paleness, cold sweat, nausea, abnormal salivation, vomiting, abdominal pain or severe headache, disturbed hearing or vision, dizziness, mental confusion, or weakness).

Additional details:

1. Use of nicotine replacement therapy. For our study, participants assigned to the control group will be given nicotine replacement therapy (combination transdermal and short-acting lozenge). In both instances, there is no link between NRT and congenital anomalies.

1a. Nicotine lozenge: In a recent trial of placebo vs. single vs. multiple medications for smoking cessation, the three most common adverse events within the lozenge group were 1) nausea: 7.8%, compared to 4.4% within placebo group, 2) mouth/throat irritation: 6.7%, compared to 3.3% within placebo group, and 3) hiccups: 6.2%, compared to 0.3% within placebo group. All other adverse events occurred <5%.

1b. Nicotine patch. The most common side effects from nicotine patch are skin irritation, insomnia, and headache or nausea. In an early but seminal placebo-controlled test of patch, there were few systemic side effects of patch use: 21% vs. 15% of smokers in the patch and placebo groups respectively reported a side effect during the treatment period. The most frequent symptoms with the patch as compared with the placebo patch were headache (4 vs. 4 percent), nausea (4 vs. 1 percent), and vertigo (4 vs. 0 percent). Transient mild itching was reported by 14% of the subjects in the patch group and 1% in those in the placebo group.

2. Use of oral nicotine pouches. ONPs are not combusted and do not contain tobacco leaf, and as a result, they deliver lower levels of toxicants than cigarettes. A recent study reported adverse events following administration of a 4mg ONP, 4mg NRT lozenge, and 4mg NRT gum. The incidence of product-related adverse events was lowest in the ONP group (12.1%) followed by NRT gum (12.5%) and NRT lozenge (30.3%). Overall, 97% of AEs were mild, with 6% of participants reporting dizziness after ONP use (the most common AE) and no reports of nausea or throat irritation following ONP use. No serious AEs were reported in the ONP group. ONP use in pregnant women has not been studied, but we expect to see similar effects as in NRT.

3. Concurrent use of cigarettes with NRT or ONPs. In a prior study from our group, large numbers of smokers concurrently smoked and used nicotine gum or other NRT products and the incidence of any significant AEs was < 1%. Recent research now demonstrates that starting nicotine patch prior to stopping smoking increases quit rates relative to starting patch at the time of cessation. With concurrent use of ONPs or traditional NRT with cigarettes, participants' nicotine intake may increase, risking nicotine overdose.

4. Use of ONPs/NRT among non-participants and non-smokers, including children. Whenever a product is given to a smoker to take home and use, there is potential that the product will be used by someone else, inclusive of non-smokers and even children. Such diversion has not been observed in an OSU Center for Tobacco Research’s ongoing R01 of e-cigarettes.

We ask established smokers exposed to tobacco and nicotine on a daily basis to sample either nicotine pouches (ONPs) or nicotine replacement therapy (NRT) products. ONPs may be less harmful to participants given the absence of tobacco. The NRT products are approved to aid in smoking cessation. Both products are available commercially. Because these products offer reduced risk and may be purchased by the participant commercially to reduce/eliminate tobacco consumption, these interventions do not represent greater than minimal risk to the participant. All other study assessments - collecting iCO data, surveys, and phone calls - are not much different than a normal quitline or clinic for smokers. As such, we do not believe this study represents greater than minimal risk to participants.

**Risk Mitigation**

While this trial is not without risk, every effort will be taken to reduce risk and undue burden on participants.

To minimize the risk of side effects from the study products:

- Participants will be provided with detailed instructions on the proper use of oral nicotine pouches and NRT.
- Participants will receive education on the potential risks and benefits of using oral nicotine pouches and NRT.
- Any adverse events or side effects experienced by participants will be documented and reported in accordance with the study protocol.

Nicotine pouches and traditional NRT do not seem to have the same potential harms of cigarettes and are available commercially; therefore, we believe participants are not at an elevated risks for sampling these products and may even benefit from the reduced tobacco intake during the switching phase. Because nicotine pouches only contain nicotine, and traditional NRT products such as lozenges and nicotine patches may help people quit or switch, we believe these products are not introducing increased harm. Knowledge gained from this trial will demonstrate best practices and the feasibility of switching to these products as a cigarette smoker, which is knowledge that is critical as these ONPs saturate the tobacco and nicotine market.

1a & 1b: Use of nicotine replacement therapy: We will exclude individuals based on standard FDA contraindications for NRT use (pregnancy, recent cardio trauma). Participants will be encouraged to contact the Study PI as soon as possible for serious AEs and for those conditions that OTC labeling suggests seeing a provider. We will withdraw participants who have a serious AE. For other AEs, the participant may be withdrawn from the study. We will also form a Data Safety and Monitoring Board. If the percent of serious or severe AEs appears to be greater than 5% this board will be notified to make a decision on early termination of the study.

2: Use of ONPs: Participants will be screened for general medical precautions and all participants will be monitored for adverse events during the study period. We will clearly advise against use of any tobacco product during pregnancy and breastfeeding and will exclude participants who are pregnant or breastfeeding. Participants will be educated about potential risks of tobacco use, including risks specific to ONPs, and concurrent use of these products with cigarettes. Any serious or unexpected adverse events will be reported to the IRB.

3. Our accompanying brochures will discuss the anticipated negative consequences of nicotine intoxication (nausea, headache) and advise participants to discontinue one or both products should they arise. We will track adverse events at every study contact and will have a toll-free number available for participants to call if they experience an adverse event (AE). All study contacts will remind participants of this number. Participants will be encouraged to contact Drs. Keller-Hamilton or Wagener as soon as possible for serious events. If they wish, they may contact their local MD. We will withdraw participants who have a serious AE, become pregnant, or begin breastfeeding. For other AEs, if the participant’s physician or the participant wishes it, the participant will be withdrawn from the study. We will assess dependence on all products used, and for participants whose dependence increases for any product without a corresponding decrease >50% in CPD, we will refer them to seek treatment for tobacco dependence.

4. Use of ONPs/NRT among non-participants and non-smokers, including children: We will strongly advise participants that they are not to share the study product with others, and that they should store the product in a secure area that is out of reach of children and pets.

5. Confidentiality: We will use the participant’s name only in the screening and participant phone calls. All other references to participant data will be under a code. All database files will include password protection to further ensure confidentiality. We do not expect this study to present greater risks than other studies our team has conducted and expect to continue treating participant data with the utmost respect and confidentiality.

# **14. Monitoring**

|  | Y | N | Comments |
| --- | --- | --- | --- |
| Does the research involve greater than minimal risk (i.e., the harms or discomforts described are beyond those ordinarily encountered in daily life or during the performance of routine physical or psychological tests)? |  |  |  |

# **15. Data Analysis**

**Internal/External Validity**

To avoid study bias in the collection of data, several measures have been implemented to enhance both internal and external validity. To address internal validity, random sampling techniques have been employed to ensure that a diverse group of participants is selected (who still meet eligibility criteria), reducing selection bias. Additionally, questions are designed to be neutral and clear, minimizing response bias.

To improve external validity, the survey is distributed to the study’s target population, ensuring the results are relevant to that population. Since participants are not anonymous, efforts are made to ensure confidentiality, reducing the risk of social desirability bias and promoting more honest responses.

**Data Analysis Techniques**

Power Analysis: Biochemically verified 7-day point prevalence abstinence from cigarettes at the end of the 12- week Switch Phase by study arm (ONP, NRT) is the primary outcome of the study and was chosen because it is a critical outcome to inform public health interventions and potential FDA regulations of ONPs. It is also stringent statistically, requiring a sample size that will provide sufficient statistical power to examine the other study outcomes. Although we will not biochemically confirm use of NRT or ONP, biochemical verification of abstinence from cigarettes combined with self-reported use of NRT or ONP is indicative of complete switching. To derive our NRT arm abstinence estimate of 13% we used our prior trial that investigated the effects of NRT sampling on cessation outcomes.^70^ Crucially, this trial included a sample of rural participants; in this rural subsample, the cessation rate was 13%.^70^ We derived our ONP arm estimate of 23% based on our preliminary data. As the goal of that study was not switching, we believe this estimate to be conservative. Assuming 80% retention at the end of the 12-week Switch Phase, a total sample of 800 participants (400 per arm), will provide greater than 95% power to detect a difference in smoking abstinence between the NRT and ONP arms for a two-sided, α=0.05 level chi-squared test. Further assuming 70% retention at the end of the 14-week Observation Phase, a sample of 700 (350 per arm) will provide over 90% power to detect a significant difference between the ONP and NRT arms.

Data Analytic Plan: Statistical analyses will be performed using SAS 9.4. P-values less than 0.05 will be considered statistically significant. Baseline demographics and smoker characteristics will be summarized by arm (ONP and NRT), as appropriate. Continuous variables will be presented as mean ± standard deviation and compared between the arms with t-tests. Categorical variables will be presented as frequencies and proportions and compared with chi-squared tests. Balance of all covariates according to randomization will be confirmed prior to conducting the below analyses. Any imbalanced covariates will be controlled for in regression models.

Aim 1: The examination of differential levels of (H1a) switching, (H1b) abstinence from cigarettes, and (H1c) cigarettes smoked per day, between the ONP and NRT study arms at the end of the 12-week Switch Phase will be the focus of Aim 1. Hypotheses 1a-b: Complete switching rates and abstinence from cigarettes will be compared between arms using logistic regression analysis, adjusting for baseline variables as needed. In an exploratory analysis, we will also evaluate whether sex modifies the associations between study group and the switching and abstinence outcomes using product interaction terms (see Section C.11). Hypothesis 1c: If needed, we will apply a normalizing transformation to the number of cigarettes smoked per day (e.g., taking the logarithm) before proceeding with the analysis as a means of removing the effects of potential outliers. We will compare cigarettes smoked per day between the two arms using linear regression analysis. The model will adjust for cigarettes smoked per day at baseline and other baseline variables if they are imbalanced.

Aim 2: The focus of Aim 2 is the examination of (H2a) product appeal and (H2b) cigarette craving and nicotine withdrawal at the end of the Switch Phase and (H2c) among complete switchers, change in cigarette dependence from baseline to the end of the Switch Phase between the study arms. Hypothesis 2a: We will examine differences in product appeal between the ONP and NRT arms at the end of the Switch Phase with linear regression models. These models will be adjusted for potential confounders including baseline variables such as age, gender, number of cigarettes smoked per day, and/or randomization stratification factors as necessary; normalizing transformations will be employed if needed. Hypothesis 2b: Similar to hypothesis 1a, the analysis of cigarette craving and withdrawal symptoms will be evaluated with linear regression models adjusting for appropriate confounders. Hypothesis 2c: Among participants who completely switched to their assigned study product, change in nicotine dependence from baseline will be compared between smoking abstainers in the ONP and NRT arms using linear regression analysis, adjusting for baseline nicotine dependence and other variables as outlined in Hypothesis 2a.

Aim 3: Aim 3 will examine differences between the ONP and NRT arms in (H3a) abstinence from cigarettes, (H3b) number of days the study products are used, and (H3c) purchase and continued use of the study products during the 14-week Observation Phase. Hypothesis 3a: The analysis of abstinence from cigarettes at the end of the Observation Phase will mirror that of hypothesis 1b and will be evaluated with a logistic regression model. Hypothesis 3b: Linear regression analysis will be used to compare the number of days the products are used during the Observation Phase between the ONP and NRT arms; a normalizing transformation will be employed if needed and appropriate baseline characteristics will be adjusted for. Hypothesis 3c: The continued purchase and use of either ONP or NRT after during the 14-week Observation Phase will be compared between the arms with logistic regression analysis, adjusting for all appropriate confounders. Missing Data: In the event of missing data, we will contact participants immediately. If a participant drops out, we will attempt to gather follow-up information by calling and trying to reach the participant through other provided contact information. However, if a participant refuses to be contacted or loses contact with the investigators, we will censor data at point of loss. Two statistical approaches will be used to handle missing data. First, we will use inverse probability weighting with propensity scores. This is a two-step procedure in which we first model the probability of missingness as a function of baseline covariates and previous outcomes. Next, the inverse of the resulting predicted probabilities (from the logistic regression model) will serve as weights in our proposed model of the response. We will compare these results to a more conservative intent-to-treat approach as a final step.

# **16. Bibliography**

1. Wilson RJ, Ryerson AB, Singh SD, King JB. Cancer Incidence in Appalachia, 2004-2011. Cancer Epidemiol Biomarkers Prev. 2016;25(2):250-258. doi:10.1158/1055-9965.EPI-15-0946
2. Yao N, Alcalá HE, Anderson R, Balkrishnan R. Cancer Disparities in Rural Appalachia: Incidence, Early Detection, and Survivorship. J Rural Health Off J Am Rural Health Assoc Natl Rural Health Care Assoc. 2017;33(4):375-381. doi:10.1111/jrh.12213
3. Arnold C. Appalachia’s Cancer Problem. J Natl Cancer Inst. 2017;109(3). doi:10.1093/jnci/djx0451.
4. County Health Rankings & Roadmaps. University of Wisconsin Population Health Institute; 2023. Accessed April 12, 2023. <https://www.countyhealthrankings.org/>
5. Centers for Disease Control and Prevention. Current Cigarette Smoking Among Adults in the United States. U.S. Department of Health and Human Services; 2023. Accessed May 5, 2023. <https://www.cdc.gov/tobacco/data_statistics/fact_sheets/adult_data/cig_smoking/index.htm#nation>
6. Nemeth JM, Liu ST, Klein EG, Ferketich AK, Kwan MP, Wewers ME. Factors Influencing Smokeless Tobacco Use in Rural Ohio Appalachia. J Community Health. 2012;37(6):1208-1217. doi:10.1007/s10900-012-9556-x
7. Meyer MG, Toborg MA, Denham SA, Mande MJ. Cultural Perspectives Concerning Adolescent Use of Tobacco and Alcohol in the Appalachian Mountain Region. J Rural Health. 2008;24(1):67-74. doi:10.1111/j.1748-0361.2008.00139.x
8. Pollard K, Jacobsen LA, Population Reference Bureau. The Appalachian Region: A Data Overview from the 2016-2020 American Community Survey Chartbook. Appalachian Regional Commission; 2022. Accessed May 2, 2023. <https://www.arc.gov/wpcontent/uploads/2022/08/PRB_ARC_Chartbook_ACS_2016-2020_FINAL_2022-09.pdf>
9. Meit M, Heffernan M, Tanenbaum E, Cherney M, Hallman V. Appalachian Diseases of Despair. Appalachian Regional Commission; 2020. Accessed May 24, 2023. <https://www.arc.gov/wpcontent/uploads/2020/11/Appalachian-Diseases-of-Despair-October-2020.pdf>
10. Levit LA, Byatt L, Lyss AP, et al. Closing the Rural Cancer Care Gap: Three Institutional Approaches. JCO Oncol Pract. 2020;16(7):422-430. doi:10.1200/OP.20.00174
11. Stevenson T, Proctor RN. The secret and soul of Marlboro: Phillip Morris and the origins, spread, and denial of nicotine freebasing. Am J Public Health. 2008;98(7):1184-1194. doi:10.2105/AJPH.2007.121657
12. Kostygina G, Ling PM. Tobacco industry use of flavourings to promote smokeless tobacco products. Tob Control. 2016;25(Suppl 2):ii40-ii49. doi:10.1136/tobaccocontrol-2016-053212
13. Curry LE, Pederson LL, Stryker JE. The changing marketing of smokeless tobacco in magazine advertisements. Nicotine Tob Res. 2011;13(7):540-547. doi:10.1093/ntr/ntr038
14. Robichaud MO, Seidenberg AB, Byron MJ. Tobacco companies introduce ‘tobacco-free’ nicotine pouches. Tob Control. 2020;29(E1):e145-e146. doi:10.1136/tobaccocontrol-2019-055321
15. Azzopardi D, Liu C, Murphy J. Chemical characterization of tobacco-free “modern” oral nicotine pouches and their position on the toxicant and risk continuums. Drug Chem Toxicol. Published online 2021:1-9. doi:10.1080/01480545.2021.1925691
16. Stanfill S, Tran H, Tyx R, et al. Characterization of Total and Unprotonated (Free) Nicotine Content of Nicotine Pouch Products. Nicotine Tob Res Off J Soc Res Nicotine Tob. Published online May 26, 2021. doi:10.1093/ntr/ntab030
17. Talbot EM, Giovenco DP, Grana R, Hrywna M, Ganz O. Cross-promotion of nicotine pouches by leading cigarette brands. Tob Control. Published online October 20, 2021:tobaccocontrol-2021-056899. doi:10.1136/tobaccocontrol-2021-056899
18. Czaplicki L, Patel M, Rahman B, Yoon S, Schillo B, Rose SW. Oral nicotine marketing claims in direct-mail advertising. Tob Control. Published online May 5, 2021:tobaccocontrol-2020-056446. doi:10.1136/tobaccocontrol-2020-056446
19. U.S. Department of Health and Human Services. The Health Consequences of Smoking—50 Years of Progress: A Report of the Surgeon General. U.S. Department of Health and Human Services, Centers for Disease Control and Prevention, National Center for Chronic Disease Prevention and Health Promotion, Office on Smoking and Health,; 2014:1-978. <https://permanent.access.gpo.gov/gpo45352/PDF> version/Full report/full-report.pdf
20. Centers for Disease Control and Prevention. Smokeless Tobacco Use in the United States. US Department of Health and Human Services, CDC; 2018. Accessed September 23, 2018. <https://www.cdc.gov/tobacco/data_statistics/fact_sheets/smokeless/use_us/index.htm>
21. Pesko MF, Robarts AMT. Adolescent Tobacco Use in Urban Versus Rural Areas of the United States: The Influence of Tobacco Control Policy Environments. J Adolesc Health. 2017;61(1):70-76. doi:10.1016/j.jadohealth.2017.01.019
22. Islami F, Bandi P, Sahar L, Ma J, Drope J, Jemal A. Cancer deaths attributable to cigarette smoking in 152 U.S. metropolitan or micropolitan statistical areas, 2013-2017. Cancer Causes Control CCC. 2021;32(3):311-316. doi:10.1007/s10552-020-01385-y
23. Lortet-Tieulent J, Goding Sauer A, Siegel RL, et al. State-Level Cancer Mortality Attributable to Cigarette Smoking in the United States. JAMA Intern Med. 2016;176(12):1792-1798. doi:10.1001/jamainternmed.2016.6530
24. Klein EG, Liber AC, Kauffman RM, Berman M, Ferketich AK. Local smoke-free policy experiences in Appalachian communities. J Community Health. 2014;39(1):11-16. doi:10.1007/s10900-013-9733-6
25. Holford TR, McKay L, Jeon J, et al. Smoking Histories by State in the U.S. Am J Prev Med. 2023;64(4 Suppl 1):S42-S52. doi:10.1016/j.amepre.2022.08.018
26. Buettner-Schmidt K, Miller DR, Maack B. Disparities in Rural Tobacco Use, Smoke-Free Policies, and Tobacco Taxes. West J Nurs Res. 2019;41(8):1184-1202. doi:10.1177/0193945919828061
27. Centers for Disease Control and Prevention. STATE System Smokefree Indoor Air Fact Sheet. U.S. Department of Health and Human Services; 2023. Accessed May 1, 2023. <https://www.cdc.gov/statesystem/factsheets/sfia/SmokeFreeIndoorAir.html#:~:text=As%20of%20December%2031%2C%202022%2C%2028%20states%20%28Arizona%2C,for%20bars%2C%20restaurants%2C%20and%20worksites%20%28private%20and%20government%29>.
28. Ozga JE, Romm KF, Turiano NA, et al. Cumulative disadvantage as a framework for understanding rural tobacco use disparities. Exp Clin Psychopharmacol. 2021;29(5):429-439. doi:10.1037/pha0000476
29. Campaign for Tobacco-Free Kids. Map of State Cigarette Tax Rates.; 2022. Accessed May 1, 2023. <https://www.tobaccofreekids.org/assets/factsheets/0222.pdf>
30. Office on Smoking and Health. STATE System Licensure Fact Sheet. National Center for Chronic Disease Prevention and Health Promotion; 2023. Accessed May 1, 2023. <https://www.cdc.gov/statesystem/factsheets/licensure/Licensure.html#anchor_1562854161>
31. Marshall JL, Thomas L, Lane NM, et al. Creating a Culture of Health in Appalachia: Disparities and Bright Spots. PDA Inc, The Cecil G. Sheps Center for Health Statistics, Appalachian Regional Commission; 2017 Accessed September 23, 2018. <https://www.arc.gov/assets/research_reports/Health_Disparities_in_Appalachia_August_2017.pdf>
32. Bolin JN, Bellamy GR, Ferdinand AO, et al. Rural Healthy People 2020: New Decade, Same Challenges. J Rural Health Off J Am Rural Health Assoc Natl Rural Health Care Assoc. 2015;31(3):326-333. doi:10.1111/jrh.12116
33. Gupta S, Scheuter C, Kundu A, Bhat N, Cohen A, Facente SN. Smoking-Cessation Interventions in Appalachia: A Systematic Review and Meta-Analysis. Am J Prev Med. 2020;58(2):261-269. doi:10.1016/j.amepre.2019.09.013
34. Nuako A, Liu J, Pham G, et al. Quantifying rural disparity in healthcare utilization in the United States: Analysis of a large midwestern healthcare system. PloS One. 2022;17(2):e0263718. doi:10.1371/journal.pone.0263718
35. Alpert HR, Koh H, Connolly GN. Free nicotine content and strategic marketing of moist snuff tobacco products in the United States: 2000-2006. Tob Control. 2008;17(5):332-338. doi:10.1136/tc.2008.025247
36. Pankow JF, Tavakoli AD, Luo W, Isabelle LM. Percent free base nicotine in the tobacco smoke particulate matter of selected commercial and reference cigarettes. Chem Res Toxicol. 2003;16(8):1014-1018. doi:10.1021/tx0340596
37. Delnevo CD, Hrywna M, Miller EJ, Wackowski OA. Examining market trends in smokeless tobacco sales in the United States: 2011-2019. Nicotine Tob Res. 2020;23(8):1420-1424. doi:10.1093/ntr/ntaa239
38. Marynak KL, Wang X, Borowiecki M, et al. Nicotine Pouch Unit Sales in the US, 2016-2020. JAMA. 2021;326(6):566-568. doi:10.1001/jama.2021.10366
39. Hrywna M, Gonsalves NJ, Delnevo CD, Wackowski OA. Nicotine pouch product awareness, interest and ever use among US adults who smoke, 2021. Tob Control. Published online February 25, 2022:tobaccocontrol-2021-057156. doi:10.1136/tobaccocontrol-2021-057156
40. Li L, Borland R, Cummings KM, et al. Patterns of Non-Cigarette Tobacco and Nicotine Use Among Current Cigarette Smokers and Recent Quitters: Findings From the 2020 ITC Four Country Smoking and Vaping Survey. Nicotine Tob Res Off J Soc Res Nicotine Tob. 2021;23(9):1611-1616. doi:10.1093/ntr/ntab040
41. Song MA, Marian C, Brasky TM, Reisinger S, Djordjevic M, Shields PG. Chemical and toxicological characteristics of conventional and low-TSNA moist snuff tobacco products. Toxicol Lett. 2016;245:68-77. doi:10.1016/j.toxlet.2016.01.012
42. Bishop E, East N, Bozhilova S, et al. An approach for the extract generation and toxicological assessment of tobacco-free ‘modern’oral nicotine pouches. Food Chem Toxicol. 2020;145:111713.
43. U.S. Food & Drug Administration. FDA Authorizes Modified Risk Tobacco Products. U.S. Food and Drug Administration; 2019. Accessed May 3, 2022. <https://www.fda.gov/tobacco-products/advertising-andpromotion/fda-authorizes-modified-risk-tobaccoproducts#:~:text=On%20October%2022%2C%202019%2C%20FDA,products%20compared%20to%20smoking%20cigarettes>.
44. Agaku IT, Ayo-Yusuf OA. The effect of exposure to pro-tobacco advertising on experimentation with emerging tobacco products among US adolescents. Health Educ Behav. 2014;41(3):275-280.
45. Brock B, Schillo BA, Moilanen M. Tobacco industry marketing: an analysis of direct mail coupons and giveaways. Tob Control. 2015;24(5):505-508.
46. Biener L, Roman AM, Mc Inerney SA, et al. Snus use and rejection in the USA. Tob Control. 2016;25(4):386-392. doi:10.1136/tobaccocontrol-2013-051342
47. Foulds J, Ramstrom L, Burke M, Fagerstrom K. Effect of smokeless tobacco (snus) on smoking and public health in Sweden. Tob Control. 2003;12:349-359.
48. Gilljam H, Galanti MR. Role of snus (oral moist snuff ) in smoking cessation and smoking reduction in Sweden: A survey of snus use in smokers and ex-smokers in Sweden. Addiction. 2003;98(9):1183-1189. doi:10.1046/j.1360-0443.2003.00379.x
49. Seidenberg AB, Ayo-Yusuf OA, Rees VW. Characteristics of “American Snus” and Swedish Snus Products for Sale in Massachusetts, USA. Nicotine Tob Res Off J Soc Res Nicotine Tob. 2018;20(2):262-266. doi:10.1093/ntr/ntw334
50. Lawler TS, Stanfill SB, Tran HT, et al. Chemical analysis of snus products from the United States and northern Europe. PLoS One. 2020;15(1):e0227837.
51. Lunell E, Fagerström K, Hughes J, Pendrill R. Pharmacokinetic Comparison of a Novel Non-tobaccoBased Nicotine Pouch (ZYN) With Conventional, Tobacco-Based Swedish Snus and American Moist Snuff. Nicotine Tob Res. 2020;22(10):1757-1763. doi:10.1093/ntr/ntaa068
52. McEwan M, Azzopardi D, Gale N, et al. A randomised study to investigate the nicotine pharmacokinetics of Oral nicotine pouches and a combustible cigarette. Eur J Drug Metab Pharmacokinet. 2022;47(2):211-221.
53. Rensch J, Liu J, Wang J, Vansickel A, Edmiston J, Sarkar M. Nicotine pharmacokinetics and subjective response among adult smokers using different flavors of on!® nicotine pouches compared to combustible cigarettes. Psychopharmacology (Berl). 2021;238(11):3325-3334. doi:10.1007/s00213-021-05948-y
54. Centers for Disease Control and Prevention. Smokeless Tobacco Product Use in the United States. U.S. Department of Health and Human Services; 2023. Accessed May 24, 2023. <https://www.cdc.gov/tobacco/data_statistics/fact_sheets/smokeless/use_us/index.htm>
55. Wyss AB, Hashibe M, Lee YCA, et al. Smokeless Tobacco Use and the Risk of Head and Neck Cancer: Pooled Analysis of US Studies in the INHANCE Consortium. Am J Epidemiol. 2016;184(10):703-716. doi:10.1093/aje/kww075
56. Food and Drug Administration. DOCKET NO. FDA-2012-N-0-0143, Harmful and Potentially Harmful Constituents in Tobacco Products; Established List; Proposed Additions; Request for Comments. Federal Register; 2019:38032-38035.
57. Soleimani F, Dobaradaran S, De-la-Torre GE, Schmidt TC, Saeedi R. Content of toxic components of cigarette, cigarette smoke vs cigarette butts: A comprehensive systematic review. Sci Total Environ. 2022;813:152667. doi:10.1016/j.scitotenv.2021.152667
58. U.S. Food & Drug Administration. FDA Authorizes Copenhagen Classic Snuff to Be Marketed as a Modified Risk Tobacco Product. U.S. Food and Drug Administration; 2023. Accessed April 26, 2023. <https://www.fda.gov/news-events/press-announcements/fda-authorizes-copenhagen-classic-snuff-bemarketed-modified-risk-tobacco-product>
59. Azzopardi D, Ebajemito J, McEwan M, et al. A randomised study to assess the nicotine pharmacokinetics of an oral nicotine pouch and two nicotine replacement therapy products. Sci Rep. 2022;12(1):6949. doi:10.1038/s41598-022-10544-x
60. Stead LF, Perera R, Bullen C, et al. Nicotine replacement therapy for smoking cessation. Cochrane Database Syst Rev. 2012;11:CD000146. doi:10.1002/14651858.CD000146.pub4
61. Carpenter MJ, Hughes JR, Gray KM, Wahlquist AE, Saladin ME, Alberg AJ. Nicotine therapy sampling to induce quit attempts among smokers unmotivated to quit: a randomized clinical trial. Arch Intern Med. 2011;171(21):1901-1907. doi:10.1001/archinternmed.2011.492
62. Carpenter MJ, Hughes JR, Solomon LJ, Callas PW. Both smoking reduction with nicotine replacement therapy and motivational advice increase future cessation among smokers unmotivated to quit. J Consult Clin Psychol. 2004;72(3):371-381. doi:10.1037/0022-006X.72.3.371
63. Burris JL, Wahlquist AE, Alberg AJ, et al. A longitudinal, naturalistic study of U.S. smokers’ trial and adoption of snus. Addict Behav. 2016;63:82-88. doi:10.1016/j.addbeh.2016.07.008
64. Carpenter MJ, Wahlquist AE, Burris JL, et al. Snus undermines quit attempts but not abstinence: a randomised clinical trial among US smokers. Tob Control. 2017;26(2):202-209. doi:10.1136/tobaccocontrol-2015-052783
65. Meier E, Wahlquist AE, Heckman BW, Cummings KM, Froeliger B, Carpenter MJ. A Pilot Randomized Crossover Trial of Electronic Cigarette Sampling Among Smokers. Nicotine Tob Res Off J Soc Res Nicotine Tob. 2017;19(2):176-182. doi:10.1093/ntr/ntw157
66. Smith TT, Koopmeiners JS, Tessier KM, et al. Randomized Trial of Low-Nicotine Cigarettes and Transdermal Nicotine. Am J Prev Med. 2019;57(4):515-524. doi:10.1016/j.amepre.2019.05.010
67. James SA, Meier EM, Wagener TL, Smith KM, Neas BR, Beebe LA. E-Cigarettes for Immediate Smoking Substitution in Women Diagnosed with Cervical Dysplasia and Associated Disorders. Int J Environ Res Public Health. 2016;13(3). doi:10.3390/ijerph13030288
68. Carpenter MJ, Wahlquist AE, Dahne J, et al. Nicotine replacement therapy sampling for smoking cessation within primary care: results from a pragmatic cluster randomized clinical trial. Addict Abingdon Engl. 2020;115(7):1358-1367. doi:10.1111/add.14953
69. Carpenter MJ, Alberg AJ, Gray KM, Saladin ME. Motivating the unmotivated for health behavior change: a randomized trial of cessation induction for smokers. Clin Trials Lond Engl. 2010;7(2):157-166. doi:10.1177/1740774510361533
70. Dahne J, Wahlquist AE, Smith TT, Carpenter MJ. The differential impact of nicotine replacement therapy sampling on cessation outcomes across established tobacco disparities groups. Prev Med. 2020;136:106096. doi:10.1016/j.ypmed.2020.106096
71. Balmford J, Borland R, Hammond D, Cummings KM. Adherence to and reasons for premature discontinuation from stop-smoking medications: data from the ITC Four-Country Survey. Nicotine Tob Res Off J Soc Res Nicotine Tob. 2011;13(2):94-102. doi:10.1093/ntr/ntq215
72. Cummings KM, Hyland A. Impact of nicotine replacement therapy on smoking behavior. Annu Rev Public Health. 2005;26:583-599. doi:10.1146/annurev.publhealth.26.021304.144501
73. Tønnesen P, Paoletti P, Gustavsson G, et al. Higher dosage nicotine patches increase one-year smoking cessation rates: results from the European CEASE trial. Collaborative European Anti-Smoking Evaluation. European Respiratory Society. Eur Respir J. 1999;13(2):238-246. doi:10.1034/j.1399-3003.1999.13b04.x
74. Garvey AJ, Kinnunen T, Nordstrom BL, et al. Effects of nicotine gum dose by level of nicotine dependence. Nicotine Tob Res Off J Soc Res Nicotine Tob. 2000;2(1):53-63. doi:10.1080/14622200050011303
75. Herrera N, Franco R, Herrera L, Partidas A, Rolando R, Fagerström KO. Nicotine gum, 2 and 4 mg, for nicotine dependence. A double-blind placebo-controlled trial within a behavior modification support program. Chest. 1995;108(2):447-451. doi:10.1378/chest.108.2.447
76. Shiffman S. Use of more nicotine lozenges leads to better success in quitting smoking. Addict Abingdon Engl. 2007;102(5):809-814. doi:10.1111/j.1360-0443.2007.01791.x
77. Gravely S, Giovino GA, Craig L, et al. Implementation of key demand-reduction measures of the WHO Framework Convention on Tobacco Control and change in smoking prevalence in 126 countries: an association study. Lancet Public Health. 2017;2(4):e166-e174. doi:10.1016/S2468-2667(17)30045-2
78. Foulds J, Veldheer S, Yingst J, et al. Development of a questionnaire for assessing dependence on electronic cigarettes among a large sample of ex-smoking E-cigarette users. Nicotine Tob Res Off J Soc Res Nicotine Tob. 2015;17(2):186-192. doi:10.1093/ntr/ntu204
79. Etter JF, Le Houezec J, Perneger TV. A Self-Administered Questionnaire to Measure Dependence on Cigarettes: The Cigarette Dependence Scale. Neuropsychopharmacology. 2003;28(2):359-370. doi:10.1038/sj.npp.1300030
80. Heatherton TF, Kozlowski LT, Frecker RC, Fagerstrom KO. The Fagerstrom Test for Nicotine Dependence: a revision of the Fagerstrom Tolerance Questionnaire. Addiction. 1991;86(9):1119-1127. doi:10.1111/j.1360-0443.1991.tb01879.x
81. Zacny JP, Conley K, Marks S. Comparing the Subjective, Psychomotor and Physiological Effects of Intravenous Nalbuphine and Morphine in Healthy Volunteers. J Pharmacol Exp Ther. 1997;280(3):1159-1169.
82. Cappelleri JC, Bushmakin AG, Baker CL, Merikle E, Olufade AO, Gilbert DG. Confirmatory factor analyses and reliability of the modified cigarette evaluation questionnaire. Addict Behav. 2007;32(5):912-923. doi:10.1016/j.addbeh.2006.06.028
83. Cox LS, Tiffany ST, Christen AG. Evaluation of the brief questionnaire of smoking urges (QSU-brief) in laboratory and clinical settings. Nicotine Tob Res. 2001;3(1):7-16. doi:10.1080/14622200020032051
84. Hughes JR. Signs and Symptoms of Tobacco Withdrawal. Arch Gen Psychiatry. 1986;43(3):289. doi:10.1001/archpsyc.1986.01800030107013
85. Webb TL, Sheeran P. Does changing behavioral intentions engender behavior change? A meta-analysis of the experimental evidence. Psychol Bull. 2006;132(2):249-268. doi:10.1037/0033-2909.132.2.249
86. Boudreaux ED, Sullivan A, Abar B, Bernstein SL, Ginde AA, Camargo CAJ. Motivation rulers for smoking cessation: a prospective observational examination of construct and predictive validity. Addict Sci Clin Pract. 2012;7(1):8. doi:10.1186/1940-0640-7-8
87. Etter JF. Financial incentives for smoking cessation in low-income smokers: study protocol for a randomized controlled trial. Trials. 2012;13:88. doi:10.1186/1745-6215-13-88
88. Tevyaw TO, Colby SM, Tidey JW, et al. Contingency management and motivational enhancement: a randomized clinical trial for college student smokers. Nicotine Tob Res Off J Soc Res Nicotine Tob. 2009;11(6):739-749. doi:10.1093/ntr/ntp058
89. Heil SH, Higgins ST, Bernstein IM, et al. Effects of voucher-based incentives on abstinence from cigarette smoking and fetal growth among pregnant women. Addict Abingdon Engl. 2008;103(6):1009-1018. doi:10.1111/j.1360-0443.2008.02237.x
90. Higgins ST, Bernstein IM, Washio Y, et al. Effects of smoking cessation with voucher-based contingency management on birth outcomes. Addict Abingdon Engl. 2010;105(11):2023-2030. doi:10.1111/j.1360-0443.2010.03073.x
91. Stoops WW, Dallery J, Fields NM, et al. An internet-based abstinence reinforcement smoking cessation intervention in rural smokers. Drug Alcohol Depend. 2009;105(1-2):56-62. doi:10.1016/j.drugalcdep.2009.06.010
92. Dunn KE, Sigmon SC, Thomas CS, Heil SH, Higgins ST. Voucher-based contingent reinforcement of smoking abstinence among methadone-maintained patients: a pilot study. J Appl Behav Anal. 2008;41(4):527-538. doi:10.1901/jaba.2008.41-527
93. Berlin I, Radzius A, Henningfield JE, Moolchan ET. Correlates of expired air carbon monoxide: effect of ethnicity and relationship with saliva cotinine and nicotine. Nicotine Tob Res Off J Soc Res Nicotine Tob. 2001;3(4):325-331. doi:10.1080/14622200110050400
94. Fritz M, Wallner R, Grohs U, Kemmler G, Saria A, Zernig G. Comparable sensitivities of urine cotinine and breath carbon monoxide at follow-up time points of three months or more in a smoking cessation trial. Pharmacology. 2010;85(4):234-240. doi:10.1159/000280435
95. Velicer WF, Prochaska JO, Rossi JS, Snow MG. Assessing outcome in smoking cessation studies. Psychol Bull. 1992;111(1):23-41. doi:10.1037/0033-2909.111.1.23
96. Jarvis MJ, Tunstall-Pedoe H, Feyerabend C, Vesey C, Saloojee Y. Comparison of tests used to distinguish smokers from nonsmokers. Am J Public Health. 1987;77(11):1435-1438. doi:10.2105/ajph.77.11.1435
97. Bedfont. iCO Smokerlyzer user manual. Published online 2015. Accessed May 16, 2023. <https://www.bedfont.com/documents/iCO-Smokerlyzer-manual.pdf>
98. Creamer MR, Wang TW, Babb S, et al. Tobacco Product Use and Cessation Indicators Among Adults —
99. United States, 2018. MMWR Morb Mortal Wkly Rep. 2019;68(45):1013-1019. doi:10.15585/mmwr.mm6845a2
100. Shiffman S, Dresler CM, Rohay JM. Successful treatment with a nicotine lozenge of smokers with prior failure in pharmacological therapy. Addict Abingdon Engl. 2004;99(1):83-92. doi:10.1111/j.1360-0443.2004.00576.x
101. Shiffman S, Dresler CM, Hajek P, Gilburt SJA, Targett DA, Strahs KR. Efficacy of a nicotine lozenge for smoking cessation. Arch Intern Med. 2002;162(11):1267-1276. doi:10.1001/archinte.162.11.1267
102. Piper ME, Smith SS, Schlam TR, et al. A randomized placebo-controlled clinical trial of 5 smoking cessation pharmacotherapies. Arch Gen Psychiatry. 2009;66(11):1253-1262. doi:10.1001/archgenpsychiatry.2009.142
103. Hughes JR, Pillitteri JL, Callas PW, Callahan R, Kenny M. Misuse of and dependence on over-thecounter nicotine gum in a volunteer sample. Nicotine Tob Res Off J Soc Res Nicotine Tob. 2004;6(1):79-84. doi:10.1080/14622200310001656894
104. Tønnesen P, Nørregaard J, Simonsen K, Säwe U. A double-blind trial of a 16-hour transdermal nicotine patch in smoking cessation. N Engl J Med. 1991;325(5):311-315. doi:10.1056/NEJM199108013250503
105. Jorenby DE, Leischow SJ, Nides MA, et al. A controlled trial of sustained-release bupropion, a nicotine patch, or both for smoking cessation. N Engl J Med. 1999;340(9):685-691. doi:10.1056/NEJM199903043400903
106. Sweeney CT, Fant RV, Fagerstrom KO, McGovern JF, Henningfield JE. Combination nicotine replacement therapy for smoking cessation: rationale, efficacy and tolerability. CNS Drugs. 2001;15(6):453-467. doi:10.2165/00023210-200115060-00004
107. Steinberg MB, Greenhaus S, Schmelzer AC, et al. Triple-combination pharmacotherapy for medically ill smokers: a randomized trial. Ann Intern Med. 2009;150(7):447-454. doi:10.7326/0003-4819-150-7-200904070-00004
108. Benowitz NL. Nicotine Safety and Toxicity. Oxford University Press, USA; 1998.
109. Pierce JP, Gilpin E, Farkas AJ. Nicotine patch use in the general population: results from the 1993 California Tobacco Survey. J Natl Cancer Inst. 1995;87(2):87-93. doi:10.1093/jnci/87.2.87
110. Rose JE, Herskovic JE, Behm FM, Westman EC. Precessation treatment with nicotine patch significantly increases abstinence rates relative to conventional treatment. Nicotine Tob Res Off J Soc Res Nicotine Tob. 2009;11(9):1067-1075. doi:10.1093/ntr/ntp103
111. Schuurmans MM, Diacon AH, van Biljon X, Bolliger CT. Effect of pre-treatment with nicotine patch on withdrawal symptoms and abstinence rates in smokers subsequently quitting with the nicotine patch: a randomized controlled trial. Addict Abingdon Engl. 2004;99(5):634-640. doi:10.1111/j.1360-0443.2004.00711.x
112. Fucito LM, Bars MP, Forray A, et al. Addressing the evidence for FDA nicotine replacement therapy label changes: a policy statement of the Association for the Treatment of Tobacco use and Dependence and the Society for Research on Nicotine and Tobacco. Nicotine Tob Res Off J Soc Res Nicotine Tob. 2014;16(7):909-914. doi:10.1093/ntr/ntu08

# **17. Appendix**

HRP-510 Appendix H - ARISE Multi-site Research
