# Supplementary material for: Testing oral nicotine pouches versus nicotine replacement therapy for cigarette harm reduction in Appalachia: The ARISE study protocol
Source: PLoS One. 2025 Dec 23;20(12):e0338503. doi: 10.1371/journal.pone.0338503 (PMC12725661; doi:10.1371/journal.pone.0338503)
Supplement: S1 Table — (DOCX) [file pone.0338503.s003.docx]

| **Supplemental Table 1.** ARISE study measures. | | | | | | |  |
| --- | --- | --- | --- | --- | --- | --- | --- |
|  | **Baseline^1^** | **Sampling Phase**   (2 weeks) | | **Switch Phase**   (12 weeks) | | **Observation**   (14 Weeks**)** |  |
| **Measure** |  | **Pre-Sampling**  **Call^2^** | **Post- Sampling Survey^3^** | **Mid- Switch Survey^4^** | **Post-Switch Survey^5^** | **Observation Survey^6^** |  |
| Sociodemographic | X |  |  |  |  |  |  |
| Socioenviroment as it pertains to tobacco use | X |  |  |  |  |  |  |
| Tobacco Use History | X |  |  |  |  |  |  |
| Stage of Change – Quitting (feelings about quitting smoking) | X |  | X | X | X | X |  |
| NRT – History of use | X |  |  |  |  |  |  |
| Smoking Abstinence – SRNT (history of quit attempts) | X |  | X | X | X | X |  |
| Questionnaire on Smoking Urges (QSU-brief; cravings) | X |  | X | X | X | X |  |
| PROMIS Nicotine Dependence (short form 4a) | X |  | X | X | X | X |  |
| Fagerstrom Test for Nicotine Dependence | X |  | X | X | X | X |  |
| Cigarette Evaluation Scale (experience using usual brand of cigarettes) | X |  | X | X | X | X |  |
| MN Nicotine Withdrawal Scale (feelings of nicotine withdrawal) | X |  | X | X | X | X |  |
| Motivation Rulers (motivation for quitting) | X |  | X | X | X | X |  |
| ATSQ (respiratory health) | X |  |  |  | X | X |  |
| Current Alcohol and Marijuana Consumption | X |  |  |  |  |  |  |
| Patient Health Questionnaire – (PHQ-2) (presence of depression symptoms) | X |  | X | X | X | X |  |
| Medications – GLP-1s | X |  | X | X | X | X |  |
| Timeline Follow-back (TLFB) - cigarettes (cigarette use in the last week) | X |  | X | X | X | X |  |
| Timeline Follow-back (TLFB) - ONP, Patch, Lozenge (ONP, patch, lozenge use in the last week) |  |  | X | X | X | X |  |
| Perceived Risk / Opinions – Cigarettes, ONPs, NRT | X |  | X | X | X | X |  |
| Current Cigarette Use |  |  | X | X | X | X |  |
| Current Smokeless Tobacco Use |  |  | X | X | X | X |  |
| Study Product Evaluation Scale – NRT (experience using NRT) |  |  | X | X | X | X |  |
| Study Product Evaluation Scale – ONP (experience using ONP) |  |  | X | X | X | X |  |
| Flavor Perceptions - ONP, Lozenge |  |  | X | X | X | X |  |
| Penn State Nicotine Pouch Dependence Scale (PSNPDI) |  |  | X | X | X | X |  |
| Penn State Nicotine Pouch Dependence Scale (modified for NRT) |  |  | X | X | X | X |  |
| Severson Dependence Scale (modified for ONP dependence) |  |  | X | X | X | X |  |
| Study Product Adherence – NRT and ONP (how well participant adhered to using study products) |  |  |  | X | X | X |  |
| Adapted Drug Effects Liking (feelings about study products) |  |  | X | X | X | X |  |
| Study Product Effects Liking (how study products make the participant feel) |  |  | X | X | X | X |  |
| Study Product 12-week Switch (how willing to permanently switch from cigarettes to study products) |  |  |  |  | X |  |  |
| Behavioral Intentions (intent to use ONP or NRT after study) |  |  |  |  | X | X |  |
| Quitting Nicotine (interest in quitting) |  |  |  |  |  | X |  |
| Product Transitions (use of ONP and NRT not provided by the study) |  |  |  |  |  | X |  |
| Adverse Events |  |  | X | X | X | X |  |
| *Confirmation of Smoking Status* | | | | | | |  |
| iCO measurement and CO questions |  | X | X | X | X | X |  |
| **^1^** Day 1  **^2^** After study kit arrives  **^3^** Prior to post-sampling call  **^4^** 6 weeks after switch date  **^5^** 12 weeks after switch date  **^6^** 14 weeks after switch date | | | | | | |  |
